# Biomimetic Scaffolds Regulating the Iron Homeostasis for Remolding Infected Osteogenic Microenvironment

**DOI:** 10.1002/advs.202407251

**Published:** 2024-10-07

**Authors:** Mengting Yin, Zhiqing Liu, Zhongyi Sun, Xinyu Qu, Ziyan Chen, Yuying Diao, Yuxuan Cheng, Sisi Shen, Xiansong Wang, Zhuyun Cai, Bingqiang Lu, Shuo Tan, Yan Wang, Xinyu Zhao, Feng Chen

**Affiliations:** ^1^ Center for Orthopaedic Science and Translational Medicine Department of Orthopaedics Shanghai Tenth People's Hospital School of Medicine Tongji University Shanghai 200072 P. R China; ^2^ Shanghai Key Laboratory of Craniomaxillofacial Development and Diseases Shanghai Stomatological Hospital & School of Stomatology Fudan University Shanghai 201102 P. R. China; ^3^ Suzhou First People's Hospital School of Medicine Anhui University of Science and Technology Anhui 232001 P.R. China; ^4^ Department of Plastic and Reconstructive Surgery Shanghai Key Laboratory of Tissue Engineering Shanghai Ninth People's Hospital Shanghai Jiao Tong University School of Medicine Shanghai 200011 P. R. China

**Keywords:** 3D printing, antibacterial activity, bone repair, in situ growth, iron homeostasis

## Abstract

The treatment of infected bone defects (IBDs) needs simultaneous elimination of infection and acceleration of bone regeneration. One mechanism that hinders the regeneration of IBDs is the iron competition between pathogens and host cells, leading to an iron deficient microenvironment that impairs the innate immune responses. In this work, an in situ modification strategy is proposed for printing iron‐active multifunctional scaffolds with iron homeostasis regulation ability for treating IBDs. As a proof‐of‐concept, ultralong hydroxyapatite (HA) nanowires are modified through in situ growth of a layer of iron gallate (FeGA) followed by incorporation in the poly(lactic‐co‐glycolic acid) (PLGA) matrix to print biomimetic PLGA based composite scaffolds containing FeGA modified HA nanowires (FeGA‐HA@PLGA). The photothermal effect of FeGA endows the scaffolds with excellent antibacterial activity. The released iron ions from the FeGA‐HA@PLGA help restore the iron homeostasis microenvironment, thereby promoting anti‐inflammatory, angiogenesis and osteogenic differentiation. The transcriptomic analysis shows that FeGA‐HA@PLGA scaffolds exert anti‐inflammatory and pro‐osteogenic differentiation by activating NF‐κB, MAPK and PI3K‐AKT signaling pathways. Animal experiments confirm the excellent bone repair performance of FeGA‐HA@PLGA scaffolds for IBDs, suggesting the promising prospect of iron homeostasis regulation therapy in future clinical applications.

## Graphical abstract

Here, an in situ modification strategy is proposed for printing iron‐active multifunctional scaffolds with iron homeostasis regulation ability for treating infected bone defects (IBDs). The FeGA‐HA@PLGA scaffold combats bacterial infection through photothermal effects in the early stage of bone infection, then helps restore the iron homeostasis microenvironment, thereby promoting anti‐inflammatory, vascularization and osteogenic differentiation.

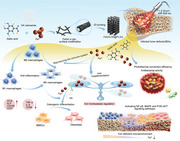

## 1 Introduction

Infected bone defects (IBDs) is a devastating complication in orthopedics and maxillofacial surgery, which usually causes persistent inflammation, delayed bone healing, and sometimes amputation or even death.^[^ ^1^ ^]^ To treat IBDs, various antibacterial agents including antibiotics, noble metal ions, and black materials that convert light to heat have been introduced to the bone scaffolds to eliminate infection.^[^ ^2^ ^]^ However, these antibacterial agents often cause long‐term safety concerns due to their toxicity at high doses and/or poor biodegradability.^[^ ^3^ ^]^ Furthermore, these antibacterial agents are inert in bioactivity, which potentially impairs the bone repair effects of the scaffolds. Therefore, multifunctional bone scaffolds with biodegradable antibacterial agents with bioactive functions such as the reconstruction of the osteogenic microenvironment are urgently required but remain challenging.

After bone injury, a profound transformation takes place within the local microenvironment, characterized by elevated levels of reactive oxygen species (ROS) and an acute inflammatory response. These phenomena significantly hinder the efficient healing process of bone defects.^[^ ^4^ ^]^ Metal‐phenolic networks (MPNs), as novel compound materials, capitalize on the intricate coordination chemistry between phenolic ligands and metal ions to exhibit robust anti‐inflammatory, antioxidant, and antibacterial properties. This represents a crucial advancement in the realm of material science.^[^ ^5^ ^]^ By synergistically combining the distinct functionalities of metallic ions and phenolic ligands, MPNs offer tailored benefits that are ideally suited to meet the specific requirements of orthopedic biomaterial applications.^[^ ^6^ ^]^ MPNs composed of bioactive metal ions (e.g., Mg^2+^,^[^ ^7^ ^]^ Fe^3+^,^[^ ^8^ ^]^ Sr^2+^,^[^ ^9^ ^]^ etc.)^[^ ^10^ ^]^ and biocompatible organic small molecule ligands (e.g., polyphenols)^[^ ^11^ ^]^ can be easily degraded under physiological environment due to their weak binding forces (i.e., coordination bonds).^[^ ^12^ ^]^ Their degradation products often provide various biological activities such as pro‐angiogenesis^[^ ^13^ ^]^ and free radical scavenging.^[^ ^14^ ^]^ Therefore, MPNs are suitable agents for designing functional scaffolds.^[^ ^15^ ^]^ Among various bioactive metal ions, iron‐based MPNs have dual functions of photothermal antibacterial activity^[^ ^16^ ^]^ and iron supplementation.^[^ ^17^ ^]^ Iron is in a delicate balanced state in the body, and it is the only hormone‐regulating trace element that responds to both the host's nutritional status and infected status.^[^ ^18^ ^]^ At the site of infection, bacteria can compete with host cells for iron to maintain their survival and proliferation,^[^ ^19^ ^]^ which leads to an imbalance in tissue iron homeostasis, thereby inhibiting the maturation of neutrophils and their immune defense capabilities.^[^ ^18^, ^20^ ^]^ One important factor for treating IBDs is iron supplementation which can reverse the iron‐deficient microenvironment to restart the immune defense function of cells in the innate immune system.^[^ ^18^, ^21^ ^]^ In addition, the iron ions can promote the transformation of proinflammatory macrophages (M1) to anti‐inflammatory macrophages (M2), ^[^ ^22^ ^]^ which promotes the regeneration process.^[^ ^23^, ^24^ ^]^ Therefore, iron‐based metal organic complex modified scaffolds are expected to have favorable treatment performance for IBDs, yet are rarely studied.

The porous architecture of the scaffolds is an appropriate microenvironment that is conducive to cell adhesion, proliferation, differentiation, and biomineralization.^[^ ^25^ ^]^ The hydroxyapatite ultralong nanowires (HA) reported in our previous work have high biocompatibility, biological activity and thermal stability,^[^ ^26^ ^]^ which stands as a preferred material for bone repair applications.^[^ ^27^ ^]^ However, due to the intrinsic brittleness of hydroxyapatite materials, it is not suitable as a base component for 3D printing.^[^ ^28^ ^]^ To improve its printability, HA is usually mixed in the polymeric materials such as poly(lactic‐co‐glycolic acid) (PLGA) for room‐temperature 3D printing that can largely protect the scaffold from thermal degradation.^[^ ^29^ ^]^

**Scheme 1:**
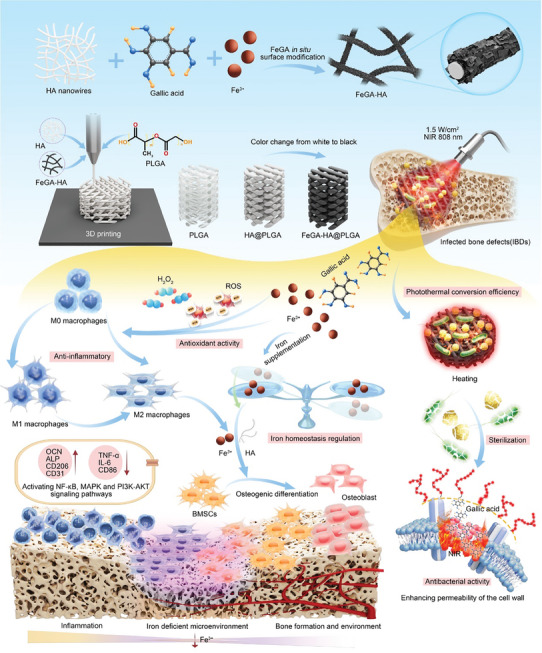
Schematic illustration of preparation and mechanism of the FeGA‐HA@PLGA scaffolds for treating IBDs.

In this work, an in situ growth strategy is proposed to prepare iron gallate (FeGA) metal organic complex modified 3D printing iron‐active multifunctional bone scaffolds with antibacterial activity, anti‐inflammatory and iron homeostasis regulation abilities using HA and PLGA as matrices (Scheme 1). The in situ growth strategy uses the minimum amount of FeGA to turn white HA nanowires into black, thereby endowing them with excellent photothermal properties while avoiding potential toxicity. The molecular dynamics (MD) simulation is used to elucidate the layered structure of FeGA on the surface of HA nanowires. The as‐obtained multifunctional scaffolds comprising FeGA modified HA nanowires (FeGA‐HA@PLGA) have designed micro‐scale porous structure, which facilitates cell adhesion, migration and growth, and sustained release of bioactive iron ions and GA molecules upon its degradation. These released iron ions can reverse the iron deficient microenvironment to reactivate the innate immune defense system in the early stages of infected bone repair. The photothermal effect endows the FeGA‐HA@PLGA scaffolds with an excellent antibacterial performance by enhancing the permeability of bacterial membranes through effective thermal simulation. The released GA molecules can scavenge ROS while the released iron ions can promote macrophages to transform to M2 phenotype. The biological activities of GA and iron ions help to transform the inflammatory microenvironment into a microenvironment conducive to tissue regeneration. By synergizing antibacterial and anti‐inflammatory activity, immune system reactivation and pro‐osteogenic differentiation properties, our FeGA‐HA@PLGA scaffolds are expected to have favorable therapeutic efficacy for IBDs.

## 2 Results and Discussion

### 2.1 The In Situ Growth FeGA‐HA Nanowires are Prepared and Characterized

**Figure 1:**
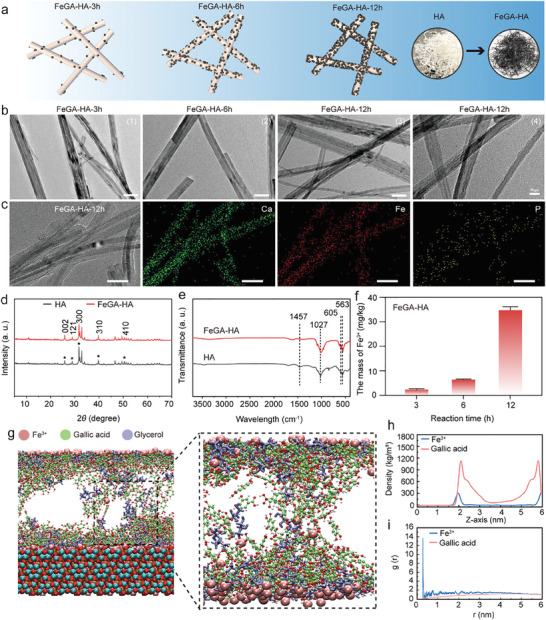
Characterization of the FeGA‐HA. a) Schematic illustration of the formation mechanism of the FeGA‐HA samples with increasing reaction time. b) TEM images of FeGA‐HA samples prepared under different reaction times: (1)–(3) Scale bar: 50 nm, (4) Scale bar: 20 nm. c) TEM image and corresponding elemental mapping images of FeGA‐HA‐12 h, Scale bar: 50 nm. d) XRD patterns of HA and FeGA‐HA. e) FTIR curves of HA and FeGA‐HA. f) The content of Fe^3+^ in the FeGA‐HA measured by ICP. g) The MD simulation results show the adsorption structure of FeGA on the HA surface. h) The relationship between the density of GA and Fe^3+^ in the system and their distance from the surface of HA. i) Radial distribution of Fe^3+^ and GA in the system before and after MD simulation.

The successful in situ growth of FeGA on the surface of HA nanowires is confirmed by a transmission electron microscope (TEM, **Figure** **1**a–c). The smooth surface of HA nanowires becomes rough with increasing reaction time. The process is accompanied by the color change of the FeGA‐HA samples from white to grey and then to black (Figure 1a; Figures S1–S3, Supporting Information). The energy‐dispersive X‐ray spectroscopy (EDS) mapping results show the uniform distribution of iron elements, proving the uniform growth of FeGA on the HA surface (Figure 1c). Compared to directly mixing FeGA nanoparticles with HA mechanically, in situ growth of FeGA on the surface of HA nanowires has several advantages including (i) ensuring uniform sustained release of iron ions and GA molecules; (ii) achieving high photothermal property at a low FeGA amount; (iii) avoiding potential toxicity caused by FeGA at a high dose. The relationship between Fe^3+^ content in the samples and reaction time is investigated. The Fe^3+^ amount in the samples prepared at 3, 6 and 12 h is determined to be 2.36, 6.05 and 34.67 mg k^−1^g by inductively coupled plasma analysis (ICP, Figure 1f). The increased Fe^3+^ content with reaction time results in the color change from white to black (Figure S2, Supporting Information). The powder X‐ray diffraction (XRD) and Fourier‐transform infrared spectroscopy (FTIR) results (Figure 1d,e) confirm the existence of HA (JCPDS 09–0432). The lack of differences observed in the XRD and FTIR results of HA and FeGA may be due to the thin and amorphous FeGA layer, which only accounts for a small proportion of the entire sample.

To investigate the binding mold between FeGA and HA, the MD simulation is conducted using a system containing Fe^3+^, GA, glycerol and HA crystals with an exposed [001] crystal plane (Figure 1g). The simulation results show that a layer of FeGA with an amorphous structure is formed on the surface of [001] crystal plane of HA. The thickness of the FeGA layer is about 2 nm (Figure 1h). The radial distribution function results show that a distinct g(r) peak appears after the aggregation reaction of Fe^3+^ and GA on the surface of HA for 50 ns (Figure 1i). The conformation analysis shows that the Fe^3+^ ions are initially adsorbed onto the HA surface followed by coordinating with carboxyl groups of GA to allow the molecular growth on the crystal plane (Figure 1g). Simultaneously, the conjugation between GA molecules and HA through the benzene rings promotes their continuous stacking, also facilitating the growth of FeGA on the crystal surface (Figures S5,S6, Supporting Information). These results preliminarily confirm that the HA surface exhibits a certain adsorption capacity for GA and Fe^3+^ ions, allowing them to aggregate and grow on the HA surface.

To further elucidate the interactions among all the molecules in the system, the binding energy of Fe^3+^, GA and glycerol to the HA surface is calculated (Figure S7, Supporting Information). The results show that Fe^3+^ ions have the strongest interaction with the HA surface with a binding energy of −397369.2±244.5 kJ mol^−1^. In contrast, the binding energy between GA and HA (i.e., −70455.9±285.5 kJ mol^−1^) is much smaller than that between Fe^3+^ and HA. The negative value of binding energy indicates the spontaneous binding reaction between HA and GA or Fe^3+^. In contrast, the binding energy between glycerol and HA is calculated to be positive (i.e., 227.5±158.8 kJ mol^−1^), indicating that there is no adsorption between glycerol and HA. The negative binding energy between Fe^3+^ and GA (i.e., −181470.7±541.2 kJ mol^−1^) indicates that the FeGA is formed during the reaction process.

### 2.2 Fabrication and Characterization of FeGA‐HA@PLGA Scaffolds

**Figure 2:**
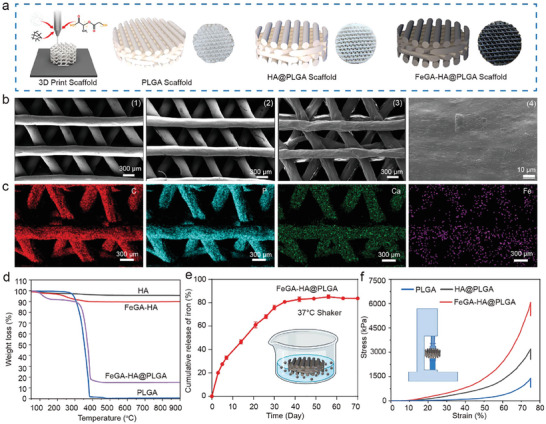
Characterization of FeGA‐HA@PLGA. a) Schematic illustration and digital photos of PLGA, HA@PLGA and FeGA‐HA@PLGA scaffolds. b) SEM images of different scaffolds of PLGA (1), HA@PLGA (2) and FeGA‐HA@PLGA (3‐4); (1–3) Scale bar: 300 µm. (4) Scale bar: 10 µm. c) EDS analysis of C, P, Ca, and Fe elements in the FeGA‐HA@PLGA scaffolds, Scale bar: 300 µm. d) The cumulative release of Fe^3+^ from the FeGA‐HA@PLGA scaffolds. e) The thermal gravimetric analysis of HA, FeGA‐HA, PLGA, and FeGA‐HA@PLGA. f) Compressive stress−strain curves of PLGA, FeGA‐HA, and FeGA‐HA@PLGA scaffolds.

3D printing technology is used to fabricate the FeGA‐HA@PLGA scaffolds with a designed porous structure using PLGA as a matrix. The PLGA with relatively fast biodegradation characteristics is beneficial for the exposure of FeGA‐HA and the Fe^3+^ release from it. The optical images of PLGA, HA@PLGA, and FeGA‐HA@PLGA 3D printing scaffolds are shown in **Figure** **2**a. The favorable printability of PLGA is not affected by the additives of HA or FeGA‐HA while the scaffold color is changed to white or black by adding HA or FeGA‐HA. The scanning electron microscope (SEM) images show that the surface of the scaffold becomes a little rough upon adding FeGA‐HA (Figure 2b). The water contact angle results show that the hydrophilicity of the FeGA‐HA@PLGA scaffolds is enhanced compared to the PLGA scaffold, which is conducive to cell adhesion and proliferation (Figure S8, Supporting Information). EDS mapping results show the uniform distribution of Fe elements, indicating that the FeGA‐HA nanowires are uniformly incorporated in the PLGA scaffold (Figure 2c). There are no characteristic diffraction peaks of FeGA that are detected due to its lower loading content and weak crystallization. This observation also suggests the effective dispersion of minute FeGA clusters on the HA (JCPDS 09–0432) surface. In addition, XRD patterns show that the FeGA‐HA addition can induce the crystallization of PLGA, which helps enhance the mechanical strength of the scaffolds (Figure S9, Supporting Information). FTIR measurements show that the typical vibrational peaks of phosphate (i.e., 1750 cm^−1^) are observed in both FeGA‐HA and FeGA‐HA@PLGA samples, also confirming the successful incorporation of FeGA‐HA in the PLGA scaffolds (Figure S10).

Iron is the fifth most abundant metal element in healthy adult bones (i.e., ≈100 ppm, less than calcium, magnesium, zinc, and strontium).^[^ ^30^ ^]^ Iron maintains bone health from multiple aspects, including participating in the metabolic process of bones, affecting calcium absorption, maintaining bone density, and ensuring bone strength.^[^ ^31^ ^]^ Iron deficiency may lead to issues such as poor bone development, slow fracture healing, and decreased bone density.^[^ ^32^ ^]^ Therefore, iron supplementation is crucial for bone repair. The amount of iron in the FeGA‐HA nanowires is determined to be ≈32 ppm by inductively coupled plasma atomic emission spectroscopy (ICP‐AES). The loading of FeGA‐HA nanowires in the FeGA‐HA@PLGA scaffolds is determined to be ≈14.4% by thermogravimetric analysis (TGA), based on which iron content in the scaffolds can be estimated to be ≈4.62 ppm (Figure 2e; Figure S11, Supporting Information). To examine the release kinetics of GA, the FeGA‐HA@PLGA scaffolds were immersed in PBS, and the absorbance of the supernatant at 259 nm was measured chronologically. The results show that ≈65% GA can be released from the FeGA‐HA@PLGA scaffolds after 23 days in a simulated physiological environment (Figures S12 and S13, Supporting Information). Benefitting from the biodegradability of PLGA, ≈80% iron can be released from the FeGA‐HA@PLGA scaffolds after 40 days in a simulated physiological environment (Figure 2d). The released iron can exert its biological effects to facilitate bone regeneration at the defect site. Besides the bioactivity, bone implants need to have a certain level of mechanical strength. Our previous study found that the ultralong HA nanowires possess high strength and high flexibility.^[^ ^27a^ ^]^ Therefore, the incorporation of HA nanowires into the PLGA scaffolds is expected to enhance its mechanical properties. The mechanical strength of PLGA, FeGA‐HA, and FeGA‐HA@PLGA scaffolds at 80% deformation are measured to be 1.4, 3.2 and 6.1 MPa (Figure 2h). The high strength of FeGA‐HA@PLGA scaffolds is attributed to the crystalline structure of the PLGA matrix and high flexibility of loaded HA nanowires. Therefore, the FeGA‐HA@PLGA scaffolds implanted into the bone defect site can provide certain mechanical support in the process of bone tissue repair.

### 2.3 The Photothermal Properties of FeGA‐HA and FeGA‐HA@PLGA

**Figure 3:**
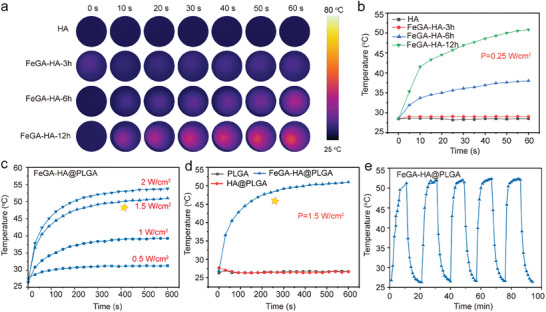
Characterization of photothermal properties. a,b) Infrared thermographic maps and photothermal heating curves of HA, FeGA‐HA‐3 h, FeGA‐HA‐6 h, and FeGA‐HA‐12 h samples under NIR laser irradiation (808 nm, 0.25 W cm^−2^). c) Photothermal heating curves of FeGA‐HA@PLGA scaffolds under NIR laser irradiations with different power densities. d) Photothermal heating curves of PLGA, HA@PLGA and FeGA‐HA@PLGA scaffolds under NIR laser irradiation (808 nm, 1.5 W cm^−2^). e) Cyclic photothermal heating/cooling curves of the FeGA‐HA@PLGA scaffolds under NIR laser irradiation (808 nm, 1.5 W cm^−2^).

Photothermal heating ability that converts electromagnetic waves into heat has been intensively used in nanomedicine.^[^ ^14^, ^33^ ^]^ For treating IBDs, high‐temperature thermal stimulation can effectively eliminate pathogenic bacteria, while mild thermal stimulation can promote bone repair.^[^ ^14a^ ^]^ Therefore, the regulation of photothermal heating temperature of bone scaffold materials is crucial for the treatment of IBDs. Our in situ modification strategy allows one to easily tune the photothermal performance of modified HA nanowires by controlling the modification layer thickness (i.e., FeGA layer). Infrared thermographic images and photothermal curves display that the FeGA‐HA‐12 h sample has the fastest heating ability with the highest peak temperature compared to FeGA‐HA‐3 h and FeGA‐HA‐6 h samples under NIR irradiation with a power density of 0.25 W cm−^2^, which is due to its highest iron content (**Figure** **3**a,b). The photothermal performances of the FeGA‐HA samples under different power densities are investigated (Figure S14, Supporting Information). These results confirm that the FeGA‐HA‐12 h sample can be rapidly heated to the antibacterial hyperthermic temperature (i.e., 49−52 °C) under a low NIR irradiation power density. Therefore, the FeGA‐HA‐12 h sample is selected to prepare the FeGA‐HA@PLGA scaffolds for further experiments. The photothermal performances of scaffolds with and without FeGA‐HA nanowires are characterized under different NIR irradiation power densities (Figure 3c). The results show that the FeGA‐HA@PLGA scaffolds is heated up to 31.1, 38.4, 49.2, and 52.1 °C under NIR irradiation for 10 min with corresponding power densities of 0.5, 1, 1.5 and 2 W cm^−^^2^. The lowest irradiation power density (i.e., 1.5 W cm^−^^2^) that can raise the temperature of the scaffold to the antibacterial temperature (i.e., 49.2 °C) is selected for subsequent experiments (Figure S15, Supporting Information). At this power density, the temperature of the FeGA‐HA@PLGA scaffolds can reach above 45 °C after irradiation for only 3 min, whereas the temperatures of the PLGA and HA@PLGA scaffolds remain below 30 °C under the same conditions (Figure 3d). Furthermore, the FeGA‐HA@PLGA scaffolds has good photothermal stability, which is proved by the cyclic heating curves (Figure 3e), and the photothermal conversion efficiency of the FeGA‐HA@PLGA is 45.9%.

### 2.4 Investigation of Antibacterial Properties with FeGA‐HA@PLGA Scaffolds

**Figure 4:**
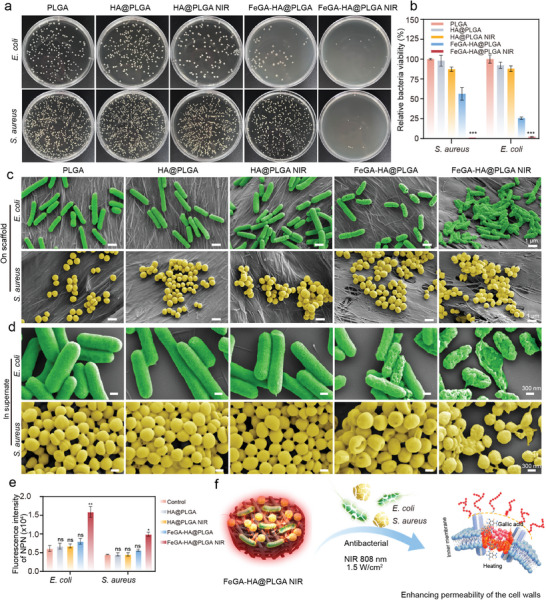
Antibacterial properties of the FeGA‐HA@PLGA scaffolds under 808 nm NIR irradiation. a) Photographs of bacterial colonies of *S. aureus* and *E. coli* treated by. b) The relative viability of bacteria treated with different groups. c,d) SEM images of *S. aureus* and *E. coli* on scaffolds or in supernate after treatment with different groups. e) Influences of different scaffolds of bacterial membrane permeability. *p < 0.05, **p < 0.01, ***p < 0.001, ****p < 0.0001. f) Schematic diagram of antibacterial mechanism of the FeGA‐HA@PLGA scaffolds under NIR irradiation.

At the infected site, bacteria proliferate rapidly and compete for iron ions from the host to ensure their survival. Therefore, it is essential not only to provide iron to the host but also to swiftly control bacterial proliferation for treating IBDs. The antibacterial activities of our scaffolds are evaluated using both Gram‐positive and Gram‐negative bacteria under an 808 nm NIR irradiation at 1.5 W cm^−^^2^. The bacterial colonies counting results show that the FeGA‐HA@PLGA NIR group has high bacterial inhibition ratios for both *S. aureus* (99.5%) and *E. coil* (99.8%) compared to other groups (**Figure** **4**a,b). The morphologies of bacteria co‐cultured with different scaffolds in supernate and on scaffolds are observed by SEM (Figure 3c,d). The bacteria with complete membranes are observed for PLGA, HA@PLGA and HA@PLGA NIR groups, indicating their low antibacterial activities, which is consistent with the bacterial colonies counting results. In contrast, the wrinkling of the bacterial membrane is observed for the FeGA‐HA@PLGA group, indicating that our FeGA‐HA@PLGA scaffolds has a certain antibacterial ability. After applying NIR irradiation, wrinkling and rupture of bacterial membranes are observed for both *S. aureus* and *E. coil* whether on the scaffolds or in the suspensions, indicating the excellent photothermal antibacterial activity of our FeGA‐HA@PLGA scaffolds. Our scaffolds possess a high heating rate at low irradiation power to achieve a photothermal antibacterial effect (47–50 °C),^[^ ^34^ ^]^ which avoids the serious irreversible thermal damage to the surrounding tissue of the defect site and adverse effects on the integration of bone implants caused by the high‐power and long‐term photothermal therapy. Simultaneously, at low irradiation power (47–50 °C) insufficient to kill bacteria, they can increase the permeability of bacterial membranes, thereby synergistically promoting the antibacterial action of antimicrobial drugs or ions.^[^ ^35^ ^]^ Therefore, to investigate the antibacterial mechanism of the FeGA‐HA@PLGA scaffolds, the bacterial membrane permeability is further examined in different treatment groups. The NPN fluorescence measurement is a typical method to evaluate the membrane integrity, where the stronger the fluorescence intensity, the worse the membrane integrity and the better the permeability. The results show that both *E.coli* and *S. aureus* show the highest fluorescence intensity in the FeGA‐HA@PLGA NIR group compared to other groups, indicating that the highest permeability of the bacterial membrane is achieved in the FeGA‐HA@PLGA NIR group (Figure 4e). The results demonstrate that the FeGA‐HA@PLGA scaffolds possess antibacterial properties, which are further enhanced under photothermal conditions. This outcome indicates that photothermal effects can increase the permeability of bacterial membranes and have a synergistic antibacterial effect with FeGA. The antibacterial mechanism could be because of the enhanced permeability of bacterial membranes caused by the photothermal effect, which enhances the antibacterial effect of FeGA‐HA@PLGA scaffolds (Figure 4f).

### 2.5 Biocompatibility, Anti‐Inflammatory and Osteogenic Differentiation Evaluation of FeGA‐HA@PLGA Scaffolds

**Figure 5:**
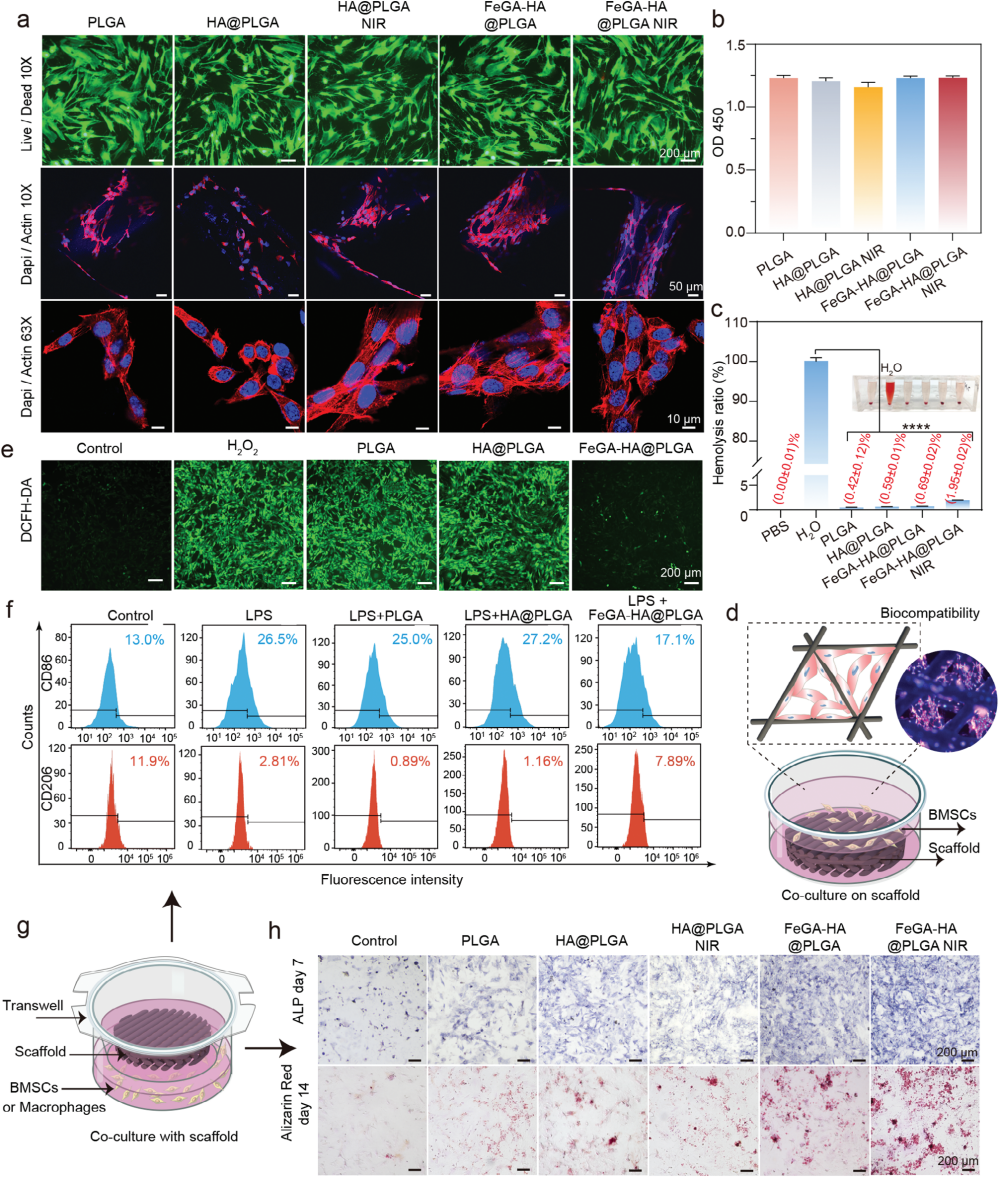
The biocompatibility, anti‐inflammatory, and osteogenic differentiation of different scaffolds in BMSCs. a) Live/Dead staining and cytoskeleton staining images (Dapi/Actin, confocal microscopy) of BMSCs cultured with different groups. Scale bar: 200 µm. b) Cytotoxicity of the different scaffolds. c) Hemolysis of rat blood treated with different scaffolds. *p < 0.05, **p < 0.01, ***p < 0.001, ****p < 0.0001. d) Schematic diagram of cell‐scaffold co‐culture models for Live/Dead and Cytoskeleton staining. e) The fluorescence images show intracellular ROS levels of BMSCs after being treated with different scaffolds. ROS was stained by DCF‐DA. Scale bar: 200 µm. f) Flow cytometry results showing CD206 and CD86 expression of macrophages with different treatments and represented as flow histograms. g) Schematic diagram of cell‐scaffold co‐culture models for ROS staining. h) ALP and ARS staining images of BMCSs co‐cultured with different groups.

A prominent advantage of 3D printing scaffolds is their designed porous structure with adjustable porosity. Such a porous structure is conducive to the growth of blood vessels and the adhesion and migration of osteogenic related cells. The biocompatibility and cell adhesion ability of our scaffolds are evaluated using bone marrow mesenchymal stem cells (BMSCs). The Live/Dead staining images and OD450 statistical analysis results indicate that all the different treatment groups have high cell viability (**Figure** **5**a,b). The laser confocal microscopy results indicate that BMSCs exhibit a healthy growth status on the scaffolds with unaffected cell morphology (Figure 5a; Figure S16, Supporting Information). Interestingly, BMSCs on the FeGA‐HA@PLGA scaffolds with or without NIR irradiation demonstrate a larger spreading area with more pseudopodia, which is an early signal for enhanced cell proliferation and differentiation. In addition, the cell adhesion density in the FeGA‐HA@PLGA scaffolds group is significantly higher than that in the unmodified scaffold group (Figure 5a). These results show that our FeGA‐HA@PLGA scaffolds has high biocompatibility. The hemolysis experiments show that all the scaffolds have good blood compatibility (Figure 5c).

Bacterial infection exacerbates the inflammatory response at the bone defect, leading to the formation of an intracellular high‐level endogenous ROS microenvironment, which is the main cause of bone necrosis in the site of IBDs.^[^ ^36^ ^]^ Therefore, it is required to eliminate the excess ROS and its assault on immune cells to transform the inflammatory microenvironment into one conducive to bone regeneration. The antioxidant component (i.e., GA) in our scaffolds can perform the task of clearing ROS. In addition, the binding of GA molecules with iron ions enhances its stability, which can contribute to the antioxidant performance of our FeGA‐HA@PLGA scaffolds. The ROS clearance capability is assessed using the DCF‐DA fluorescence probe (Figure 5d). H_2_O_2_ stimulation alone serves as the positive control and the untreated group serves as the negative control. The fluorescence images show that the ROS fluorescence intensity in BMSCs treated with the FeGA‐HA@PLGA+H_2_O_2_ is significantly lower than that in the PLGA+H_2_O_2_ or HA@PLGA+H_2_O_2_ groups, indicating that the FeGA‐HA@PLGA scaffolds has outstanding ROS scavenging ability (Figure 5e). In addition, the ROS scavenging performances of the scaffold against other different free radicals (such as DPPH, ABTS, PTIO) are also evaluated (Figures S17–S19, Supporting Information). All these results confirm the excellent antioxidant properties of our FeGA‐HA@PLGA scaffolds.

The anti‐inflammatory properties of our FeGA‐HA@PLGA scaffolds are not only achieved by scavenging ROS, but also by supplementing iron ions into the inflammatory microenvironment. The released iron ions can reverse the iron deficient microenvironment caused by bacterial proliferation, thereby activating the innate immune response suppressed by iron deficiency, and promoting the differentiation of macrophages toward the anti‐inflammatory M2 phenotype. This differentiation can be observed by measuring macrophage markers (e.g., CD86 for M1 macrophages and CD206 for M2 macrophages). As shown in Figure 5f, the percentages of M1 macrophages are measured to be 26.5%, 25.0%, 27.2%, and 17.1% for the lipopolysaccharide (LPS), PLGA‐LPS, HA@PLGA‐LPS, and FeGA‐HA@PLGA‐LPS groups. In contrast, the M2 macrophage percentages in these groups show an opposite trend where the FeGA‐HA@PLGA+LPS group has the highest M2 macrophage content (i.e., 7.9%). These results confirm the efficacy of FeGA‐HA@PLGA in alleviating the inflammatory reaction, which is conducive to maintaining immune balance, combating inflammatory responses, and inducing tissue repair.

An ideal orthopedic implant scaffold material should have favorable osteogenesis and bone induction properties.^[^ ^37^ ^]^ After completing the anti‐inflammatory process, the next important step of the scaffolds is to promote osteogenic differentiation. The alkaline phosphatase (ALP) staining and alizarin red staining of BMSCs are commonly used to investigate their osteogenic differentiation status. The ALP activity and calcium deposition are evaluated for different scaffolds using a Transwell culture method (Figure 5g). After 7 days of cultivation, the FeGA‐HA@PLGA group shows increased ALP activity compared to the PLGA and HA@PLGA groups, indicating that the FeGA‐HA@PLGA scaffolds can promote proliferation and differentiation of BMSCs (Figure 5h; Figure S20, Supporting Information). Interestingly, the ALP content is further enhanced when NIR irradiation is applied, which implies that moderate thermal stimulation is also beneficial for the proliferation and differentiation of BMSCs. The deposition of calcium phosphate is considered a hallmark of bone regeneration. The alizarin red staining results show that both FeGA‐HA@PLGA and FeGA‐HA@PLGA NIR groups have pronounced calcium deposition (i.e., red regions), indicating that the BMSCs in these two groups are undergoing osteogenic differentiation or have already differentiated into osteoblasts. These results confirm the outstanding osteogenic efficacy of our FeGA‐HA@PLGA scaffolds, especially under NIR irradiation. The outstanding pro‐osteogenesis of the FeGA‐HA@PLGA scaffolds is attributed to the coupled factors including (i) the regulation of iron homeostasis by the released iron ions, (ii) the excellent anti‐inflammatory effects of GA molecules, and (iii) the mild thermal stimulation generated by the FeGA photothermal agent.

### 2.6 Mechanism Exploration of Anti‐Inflammatory and Osteogenic‐Related of FeGA‐HA@PLGA Scaffolds

**Figure 6:**
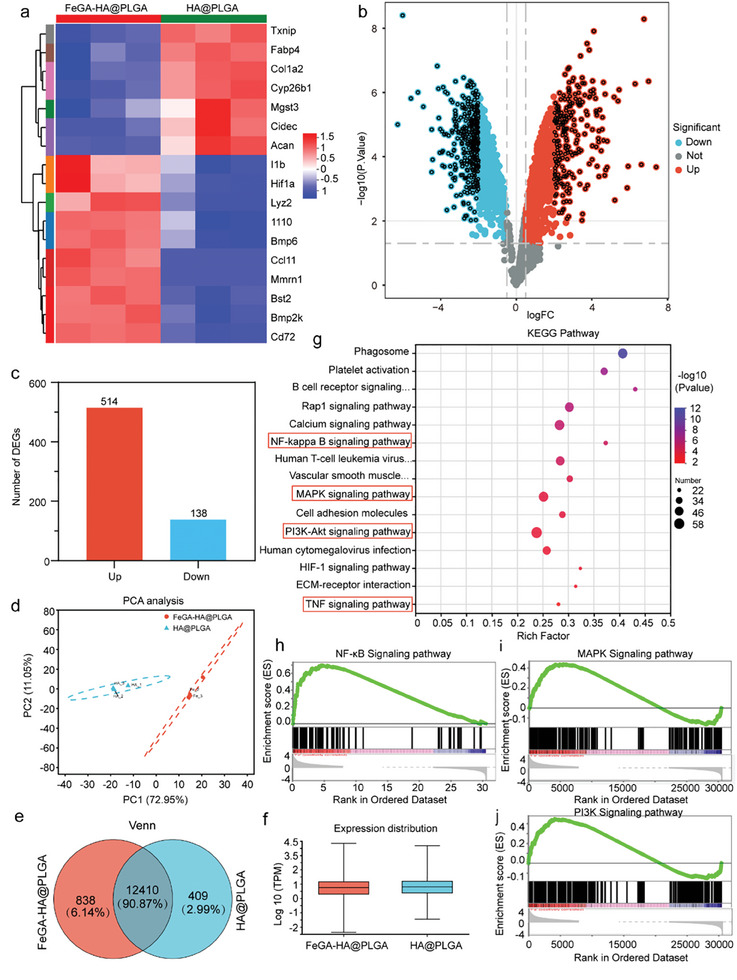
Transcriptome sequencing analysis of BMSCs co‐cultured with HA@PLGA or FeGA‐HA@PLGA scaffolds. a) Hot map of genes related to inflammation and osteogenesis for HA@PLGA and FeGA‐HA@PLGA groups (n = 3). b) Volcano plot showing differently expressed genes of the HA@PLGA and FeGA‐HA@PLGA groups. c) The number of upregulated and downregulated genes. d) Principal component analysis (PCA) suggesting the variance between the HA@PLGA and FeGA‐HA@PLGA groups. e) Venn diagram for the obtained genes and miRNAs. f) Box plot depicting TPM values. g) Pathway enrichment of different genes involved in the immune system from gene ontology analysis. h–j) Gene set enrichment analysis of anti‐inflammatory and osteogenesis‐related signaling pathways.

To gain insights into the anti‐inflammatory and pro‐osteogenesis mechanism of FeGA‐HA@PLGA scaffolds, the RNA transcriptome sequencing (RNA‐seq) of BMSCs co‐cultured with HA@PLGA or FeGA‐HA@PLGA scaffolds are measured and analyzed (**Figure** **6**). The bioinformatics analysis shows that there is a total of 652 differentially expressed genes (DEGs) including 514 upregulated genes and 138 downregulated genes by screening tens of thousands of genes for the two groups of HA@PLGA and FeGA‐HA@PLGA (Figure 6c). Among these DEGs, some can be classified as osteogenesis related DEGs including inflammation regulation (e.g., IL‐10, Bst2, and Cd72), osteogenic differentiation (e.g., Bmp6 and Bmp2k) and vascular formation (e.g., Hif‐1a), which shows an upregulation in FeGA‐HA@PLGA group and a downregulation in HA@PLGA group (Figure 6a,b). Quantitative analysis of gene expression distribution in various samples using TPM (Transcripts Per Million), and the findings indicated that there was no significant batch effect observed between the two groups of samples (Figure 6f). Furthermore, PCA revealed the differences in gene expression variability in BMSCs when comparing cultures with and without the application of FeGA, specifically between the control group and the group treated with HA@PLGA and FeGA‐HA@PLGA. (Figure 6d). Conducting a Venn analysis between HA@PLGA and FeGA‐HA@PLGA revealed 12410 genes that are co‐expressed. The results indicate the presence of shared and uniquely expressed genes between the two groups. In addition, the effects of FeGA‐HA@PLGA scaffolds on the biological process (BP), cellular component (CC), and molecular function (MF) in BMSCs can be gained using the gene ontology (GO) analysis. The GO analysis results show that DEGs in the FeGA‐HA@PLGA group are enriched in numerous specific function‐related BPs (e.g., immunity regulation, T cell‐mediated immunity, and angiogenesis promotion), CC (e.g., extracellular collagen and cell adhesion molecules) and MF (e.g., cytoskeletal protein binding, growth factor binding and signaling receptor binding) (Figure S21, Supporting Information). These BP, CC or MF are guided by several signaling pathways that are closely associated with immunity regulation and osteogenic differentiation including KEGG, NF‐κB, PI3K‐AKT and MAPK pathways (Figure 6g). FeGA induced modifications in cellular constituents and molecular functions, encompassing the extracellular matrix, regions rich in collagen, and the regulation of signaling receptor activities. Specifically, the KEGG PI3K‐AKT and MAPK pathways are related to osteogenesis and the NF‐κB pathway is noted by its regulation of the immune response, inflammation and cell survival. PI3K‐AKT is also an important intracellular pathway that can regulate cell survival, proliferation and protein synthesis. Among these pathways, PI3K‐AKT and NF‐κB, which are involved in osteogenesis and anti‐inflammatory, were notably affected by FeGA. In addition, the GO analysis results of HA@PLGA, FeGA‐HA@PLGA, and FeGA‐HA@PLGA NIR also show that mild heat stimulation can promote cell proliferation differentiation and biological regulation (Figure S21–S24, Supporting Information). Western blot analysis shows increased activity of PI3K‐AKT and MAPK signaling pathways and decreased activity of NF‐κB signaling pathway upon treatment of FeGA‐HA@PLGA, which is consistent with the KEGG enrichment results (Figure S25, Supporting Information). Moreover, gene set enrichment analysis (GSEA) found that the immunomodulatory proteins of Tnfrsf11a and Tlr4 were up‐regulated, indicating that FeGA‐HA@PLGA scaffolds may exhibit a positive influence on immunomodulation by up‐regulating the response of BMSCs to the iron homeostasis regulation (Figure 6h–j). Intriguingly, the proteins PIK3CG, VEGFA, and VEGFC were identified as being significantly upregulated in processes that pertain to cellular reactions to mechanical stimuli, hinting at a possible cooperative influence of these pathways when interacting with FeGA‐HA@PLGA. These results reveal that the FeGA‐HA@PLGA scaffolds can participate in the activation of processes pertinent to inflammatory regulation, angiogenesis promotion and pro‐osteogenic differentiation, to transform an infected microenvironment into one conducive to bone regeneration.

### 2.7 Performance of FeGA‐HA@PLGA Scaffolds in Promoting Bone Regeneration

**Figure 7:**
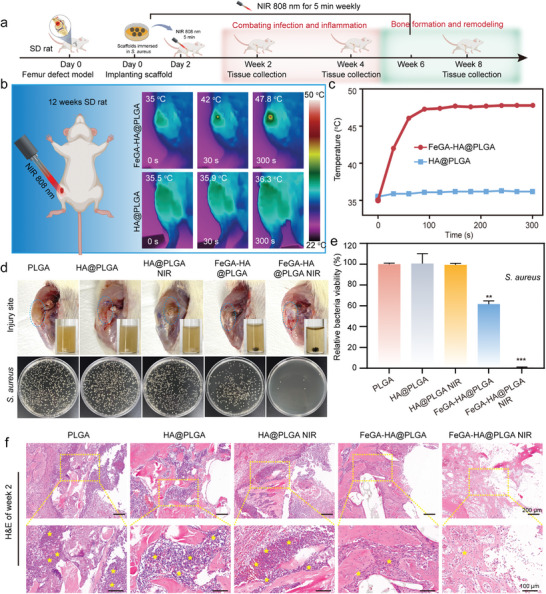
Characterization of in vivo antibacterial ability of FeGA‐HA@PLGA scaffolds. a) Schematic illustration of the animal experiment timeline. b, c) Thermal graphics (b) and temperature changes (c) of implant sites for the HA@PLGA and FeGA‐HA@PLGA groups under NIR irradiation (808 nm, 1.5 W cm^−2^). d) Top: photographs of the implant sites and media cultured with the implanted scaffolds; Bottom: photographs of bacterial colonies co‐cultured with different scaffolds. e) In vivo antibacterial efficiency of different implanted scaffolds. *p < 0.05, **p < 0.01, ***p < 0.001, ****p < 0.0001. f) The H&E staining images show the degree of infection in the soft tissues and bone tissues surrounding the implants (yellow stars represent the neutrophil cells).

The infected femoral bone defect model using SD rats is used to evaluate the efficacy of the FeGA‐HA@PLGA scaffolds for treating IBDs. Antimicrobial analysis, osteogenic analysis and immunohistochemical analysis are performed at the time points of 2 weeks, 4 weeks, and 8 weeks (**Figure** **7**a). The implantation sites of HA@PLGA NIR and FeGA‐HA@PLGA NIR groups are irradiated with 808 nm NIR (the power density is 1.5 W/cm^2^) for 5 min once a week after implantation (Figure 7b). As shown in Figure 7b,c, the temperature of the FeGA‐HA@PLGA scaffolds is rapidly increased to 42.0 °C within 30 s then further to 47.8 °C within 300 s under the NIR irradiation, while the temperature of the HA@PLGA scaffold shows no significant change under the same condition. These results show that the FeGA‐HA@PLGA scaffolds maintain their excellent photothermal effects when implanted into the femoral bone defect. Intervention for bone infection must be initiated in the early stages of infection. Otherwise, the infection may spread to surrounding tissues and form local infection foci, which could lead to more severe symptoms, such as suppuration, abscess formation, local exudation, and even functional impairment. Early infection inhibition effects of scaffolds are evaluated by retrieving and further in vitro co‐culturing the implanted scaffolds with broth medium. As shown in Figure 7d, the yellowing of the muscle tissues with secretion and suppuration at the implantation sites for PLGA, HA@PLGA, and HA@PLGA NIR groups can be visually observed. In contrast, the FeGA‐HA@PLGA group shows minimal yellowing and suppuration. And it becomes even better when the NIR irradiation is applied. These observations are consistent with the turbidity of the culture mediums co‐cultured with the extracted implants. The culture medium co‐cultured with the FeGA‐HA@PLGA NIR group is clear while culture media co‐cultured with other groups become turbid, indicating that there are bacterial residues on the scaffolds of these groups. The bacterial concentration is measured using the agar plate counting method (Figure 7d,e). The results show that the FeGA‐HA@PLGA scaffolds has a certain antibacterial effect (i.e., ≈60%) that can be largely enhanced under NIR irradiation (i.e., ≈100%). While the other groups have no antibacterial effects (i.e., ≈0%). These in vivo results confirm the excellent photothermal antibacterial capability of our FeGA‐HA@PLGA scaffolds.

**Figure 8:**
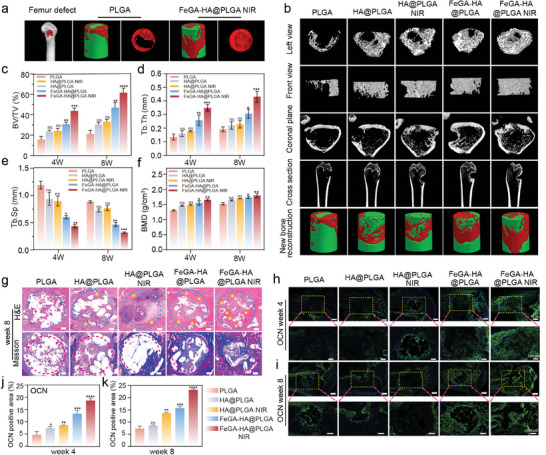
Bone regeneration characterization of FeGA‐HA@PLGA scaffolds for treating IBDs. a) 3D reconstruction images showing the formation and distribution of new bones (highlighted in red). b) Micro‐CT images from different views and three‐dimensional reconstruction images for different groups at Week 4 and 8 after surgery. c–f) Quantitative analysis of bone volume/tissue volume (BV/TV), trabecular thickness (Tb. Th), bone trabecular separation (Tb. Sp) and bone mineral density (BMD) in defect sites at Week 4 and 8 after surgery. g) H&E staining and Masson trichrome staining of the scaffold implantation sites. The yellow stars indicate the distribution of new bone. h,i) Immunofluorescence staining of OCN (green) at Week 4 and 8 after scaffold implantation. j,k) Quantitative expression of OCN. *p < 0.05, **p < 0.01, ***p < 0.001, ****p < 0.0001.

**Figure 9:**
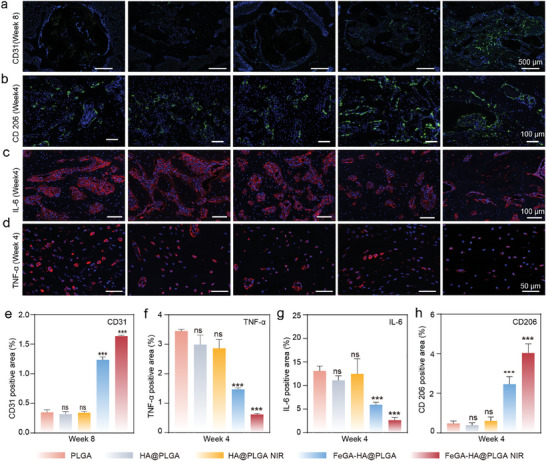
Images of Immunofluorescence staining images of a) CD31, b) CD206, c) IL‐6, and d) TNF‐α. e–h) Quantitative expression of CD31, CD206, IL‐6, and TNF‐α. *p < 0.05, **p < 0.01, ***p < 0.001, ****p < 0.0001.

To investigate the infection status at the implantation site, H&E staining is performed on the harvested samples taken at the time point of week 2. The H&E staining results reveal that inflammatory cells that gather at the defect site in FeGA‐HA@PLGA and FeGA‐HA@PLGA NIR groups are much fewer than those in the PLGA, HA@PLGA, and HA@PLGA NIR groups (indicated by yellow stars), confirming that the FeGA‐HA@PLGA scaffolds can attenuate early inflammatory responses in the treatment for IBDs. The anti‐inflammatory effects of the FeGA‐HA@PLGA scaffolds could be attributed to its iron homeostasis regulation as well as its excellent antibacterial effects. The bone repair performance of our FeGA‐HA@PLGA scaffolds is characterized by assessing scaffold degradation and the formation of new bone at the defect sites using various methods including Micro‐CT, histological staining, and immunohistochemical staining (Figures 8, 9). The reconstructed three‐dimensional images of the regenerated femur at week 8 are shown in **Figure** **8**a,b (the red region represents new bone and the green region represents the scaffold). It is evident that the FeGA‐HA@PLGA NIR group has a higher new bone density at the defect site compared to the HA@PLGA group. The micro‐CT images from different views and three‐dimensional reconstruction images show the information on new bone formation at the defect sites (Figure 8b). A large amount of new bone can be observed from edge to center in the FeGA‐HA@PLGA NIR group. In contrast, the new bone amount in other groups is less than that in the FeGA‐HA@PLGA NIR group. The ranking of new bone amount observed by Micro‐CT is PLGA < HA@PLGA < HA@PLGA NIR < FeGA‐HA@PLGA < FeGA‐HA@PLGA NIR. The quantitative analysis results are consistent with the Micro‐CT images. Among all the groups, the values of trabecular thickness (Tb. Th), bone volume/tissue volume (BV/TV) and bone mineral density (BMD) in the FeGA‐HA@PLGA NIR group are the highest, while the value of bone trabecular separation (Tb. Sp) in FeGA‐HA@PLGA NIR group is the lowest, demonstrating the best bone repair performance of the FeGA‐HA@PLGA NIR group (Figure 8c–f). The excellent bone repair performance is mainly due to the reconstruction of the bone defect microenvironment via iron homeostasis regulation combined with moderate photothermal therapy.

In the H&E staining results, the defect areas are indicated by blue dashed circles, while the locations of new bone are denoted by pink regions marked with yellow stars. Upon examination of the first row of Figure 8g, it becomes evident that the defect area progressively diminishes from left to right. Notably, the PLGA group exhibits the largest defect area, whereas the FeGA‐HA@PLGA NIR group displays the smallest defect area. In the Masson staining, collagen and mineralized bone are visualized by the blue color, as shown in Figure 8g. The results indicate that the FeGA‐HA@PLGA NIR group exhibits the highest deposition of collagen and mineralized bone area. The FeGA‐HA@PLGA group without NIR irradiation has the second most collagen deposition and mineralization, which is much more than the other groups (PLGA, HA@PLGA, HA@PLGA NIR). As shown in Figures S26,S27 (Supporting Information), the H&E and Masson staining results collected in Week 4 maintain a similar trend to that in Week 8. These results are consistent with in vitro osteogenic differentiation results (Figure 5h), demonstrating that the FeGA‐HA@PLGA scaffolds combined with mild thermal stimulation can promote osteogenic differentiation both in vitro and in vivo.

Excessive inflammatory response can hinder osteogenesis, eventually resulting in delayed bone regeneration or even implant failure. Macrophage's marker for M2 polarization is closely related to anti‐inflammatory and tissue repair, which promotes the repair and regeneration of IBDs. To investigate the in vivo antioxidant and immunomodulatory characteristics of the FeGA‐HA@PLGA scaffolds, immunohistochemistry assays are conducted to assess the expression of CD206, TNF‐α and IL‐6, while immunofluorescence staining is performed to identify macrophage markers (e.g., CD206 as a marker for M2 phenotype). The results show that the expression levels of TNF‐α and IL‐6 are quite high for the PLGA, HA@PLGA, and HA@PLGA NIR groups but they are very low for FeGA‐HA@PLGA groups with or without NIR irradiation (**Figure** **9**b,c). The quantitative results are consistent with the fluorescent staining results (Figure 9f,g). The CD206 (green color) immunofluorescence images show that the macrophages in all groups are predominantly in anti‐inflammatory (M2) phenotype where the amounts of CD206 in the FeGA‐HA@PLGA group is significantly higher than that in other groups, showing the anti‐inflammatory effect of FeGA‐HA@PLGA. The quantitative results of CD206 are consistent with the fluorescent staining results (Figure 9b). It is worth mentioning that a mild immune response is also essential to fight bacterial infection and promote osteogenic differentiation of BMSCs. In the early stage of IBDs, the FeGA‐HA@PLGA NIR group can effectively eliminate bacteria due to its excellent photothermal properties. The slowly released iron ions accelerate the immune response by regulating iron homeostasis, thereby promoting the M2 polarization of macrophages that is beneficial to osteogenesis. These results are consistent with the expression levels of pro‐inflammatory and anti‐inflammatory genes in the RNA‐seq results.

To further investigate the effects of the FeGA‐HA@PLGA NIR group on angiogenesis and osteogenesis, CD31 and OCN as the representative markers for angiogenesis and osteogenic differentiation are investigated using immunofluorescent histochemical staining. As shown in Figure 8j,k, the OCN expression level of the FeGA‐HA@PLGA group is significantly higher than that of the other groups at both Week 4 and Week 8, which is further enhanced by applying NIR irradiation, indicating the osteogenic differentiation effects of the FeGA‐HA@PLGA scaffolds and the mild thermal stimulation. In addition, immunofluorescence staining results show that the expression of CD31 in the FeGA‐HA@PLGA NIR group is much higher than that in other groups (Figure 9a), proving its pro‐angiogenic effects, which is essential for osteoblast function, encompassing proliferation, differentiation, and bone formation.^[^ ^38^ ^]^ The quantitative results of CD31 and OCN are consistent with the fluorescent staining results (Figures 9e and 8h,i). After eight weeks of treatment, important organs are collected for histological examination using H&E staining. No pathological abnormalities are observed in all the groups (Figure S28, Supporting Information), suggesting that all the scaffolds have good biocompatibility in vivo.

## 3 Conclusions

In this work, we report on an in situ modification strategy for preparing iron‐active multifunctional scaffolds with photothermal antibacterial activity, and anti‐inflammatory and iron homeostasis regulation abilities for infected bone regeneration. The strategy involves the initial in situ growth of a layer of FeGA on the HA ultralong nanowires and the subsequent incorporation of the modified nanowires in the PLGA matrix for 3D printing. The as‐prepared FeGA‐HA@PLGA scaffolds can effectively eliminate bacterial infection through its excellent photothermal effects. Additionally, the released iron ions can regulate dynamic iron homeostasis, reactivate the innate immune system and promote the polarization of macrophages towards the M2 phenotype, thereby transforming the infected microenvironment into one conducive to bone regeneration. The animal experiments confirm that the FeGA‐HA@PLGA scaffolds significantly promotes the formation of new bone through combined antibacterial, anti‐inflammatory, pro‐angiogenic, and osteogenic effects. The transcriptomic analysis shows that FeGA‐HA@PLGA scaffolds exert anti‐inflammatory and pro‐osteogenic differentiation by activating NF‐κB, MAPK and PI3K‐AKT signaling pathways. In summary, this study provides a prospective strategy for the regeneration of IBDs through the regulation of iron homeostasis by combining biomimetic microarchitecture and iron‐active ions, and this iron‐active multifunctional scaffold provides broad implications for bone defect repair applications.

## 4 Experimental Section

### Fabrication of HA and FeGA‐HA

The ultra‐long hydroxyapatite (HA) nanowires were synthesized using this previous method.^[^ ^26a^ ^]^ In a typical experiment, 10 mL of CaCl_2_ (22 g) aqueous solution and 10 mL of NaOH (7 g) solution were each added into a mixture of water (9 mL), methanol (4 mL), and oleic acid (7 mL) under mechanical agitation (300 r min^−1^). Next, 10 mL of NaH_2_PO_4_·2H_2_O (2.88 g) solution was introduced into the previous mixture under mechanical agitation (300 r min^−1^). The resulting mixture was then transferred to a 100 mL vessel for solvothermal reaction at 180 °C for 24 h. Upon cooling the reaction system to room temperature, the solvothermal product slurry containing ultralong HA nanowires was obtained. The ultralong HA nanowires were prepared by dispersing the solvothermal product slurry in absolute ethanol. After separation, the nanowires underwent three rounds of washing with ethanol and deionized water, before being dispersed once again in ethanol for future utilization. Iron gallate (FeGA) was grown in situ on the surface of HA using a solvothermal method. 1 mL of 0.1 M Fe(NO_3_)_2_·9H_2_O and 1 mL of 0.3 M gallic acid (GA) were added to 13 mL of glycerol and stirred until dissolved. The pH was adjusted to 9 with sodium hydroxide, followed by agitation for 30 min and the addition of 0.02 g of HA. The mixture was transferred to a 100 mL vessel for solvothermal reaction at 180 °C for 24 h. Finally, the iron gallate nanowires (FeGA‐HA) were purified with anhydrous ethanol and dried at 60 °C.

### Characterization of HA and FeGA‐HA

The morphological characterization of HA and FeGA‐HA was examined by transmission electron microscope (TEM) with a JEOL JEM 2100F. The X‐ray diffraction (XRD) patterns of HA and FeGA‐HA were acquired using the Empyrea instrument (Rigaku Smartlab 9KW, Japan), while Fourier transform infrared (FTIR) spectra were obtained via an FTIR spectrometer (Thermo Scientific Nicolet iS20, USA). The HA and FeGA‐HA were captured by a microscope.

### The Molecular Dynamics of FeGA‐H

Calcium ion‐rich HA [001] surfaces were constructed respectively, and the lengths of the HA [001] crystal model in the XYZ direction were 6.2 nm × 8.0 nm × 22.0 nm respectively. Fill the simulation box with 900 gallic acid molecules, 300 Fe^3+^ ions, 300 glycerol molecules, and counter ions respectively. The MD simulation uses the Gromacs 2019.6 program^[^ ^39^ ^]^ and was performed under constant temperature, constant pressure, and periodic boundary conditions. The Charmm 36 all‐atom force field was used for small molecules,^[^ ^40^ ^]^ while the INTERFACE force field was utilized for HAP molecules.^[^ ^41^ ^]^ Parameters for the force fields were sourced from existing literature. During the MD simulation, all hydrogen bonds involved were constrained using the LINCS algorithm, and the integration step was 2 fs. Electrostatic interactions were calculated using the (Particle‐mesh Ewald) PME method.^[^ ^42^ ^]^ Finally, a 50 ns MD simulation was performed on the composite object, and the conformation was saved every 10 ps. The visualization of the simulation results was completed using the Gromacs embedded program and VMD.

### 3D Printing of PLGA, HA@PLGA, and FeGA‐HA@PLGA

A 3D porous composite scaffold was created with the assistance of a 3D biological printer (Regenovo 3D Bio‐Architect Work Station, which was purchased from Regenovo) under the supervision of a supporting computer workstation. Before 3D printing, 0.15 g of HA or FeGA‐HA were dispersed in 15 mL of 1,4‐Dioxane and stirred to form uniform dispersion, respectively. Subsequently, 0.35 g of PLGA (85:15 lactide to glycolide ratio, Mw 120000, from Shandong Institute of Biomaterials) was added to the aforementioned mixed solution and stirred overnight. Then, the product was evaporated for 1 hour to remove 1,4‐Dioxane. Finally, computer‐aided design (CAD) software was employed for the 3D modeling of scaffold materials. A cylindrical structure with a diameter of 3 mm and a height of 4 mm was designed, configuring the material properties to a filament diameter of 350 µm and a porosity of 60%.

### Characterization of PLGA, HA@PLGA, and FeGA‐HA@PLGA

The morphological characterization of the scaffolds was examined by scanning electron microscopy (SEM) with a ZEISS Gemini SEM 300. The XRD patterns of the scaffolds were acquired using the Empyrea instrument (Rigaku Smartlab 9KW, Japan), while FTIR spectra were obtained via an FTIR spectrometer (Thermo Scientific Nicolet iS20, USA). The content of FeGA‐HA within the FeGA‐HA@PLGA scaffolds was assessed using thermogravimetry (Rigaku, Japan).

### Thermogravimetry Analysis

HA, FeGA‐HA, PLGA, and FeGA‐HA@PLGA were performed on a thermogravimetric analyzer. The temperature range for this study was from room temperature to 900 °C. The furnace heating rate was 5 °C·min^−1^ with a controlled mass flow of air of 60·mL min^−1^ as reagent gas. The sample mass placed in a scaffold inside the balance was around 10 mg for each analysis. Subsequently, the thermogravimetry (TG) curves were analyzed.

### In Vitro Fe Ions Release Measurement

The scaffolds were immersed in 3 mL of PBS (pH 7.4) in a shaker (37 °C, 100 rpm) for 1, 3, 5, 7, 14, 21, 28, and 70 days. The release of Fe ions was quantified by using an inductively coupled plasma source spectrometer (Prodigy‐ICP, USA).

### In Vitro GA Release Measurement

To determine the GA release profile, five scaffolds of FeGA‐HA@PLGA were put in 2 mL PBS. The supernatant was taken for absorbance measurements at 259 nm using a UV spectrometer (UV‐5100, Shanghai, China) at different times and an identical volume of fresh PBS solution was added to the release system. Then, the corresponding concentrations of the GA in the supernatant were calculated from the absorbances using the calibration curve (Figure S12, Supporting Information). The release amount was determined using the following equation:

(1) $$Release\left(\%\right)=C_{t}/M_{0}\times 100\%$$

where C_t_ refers to the calculated concentration of GA in the supernatant at t day and M_0_ refers to the mass of GA loaded in the FeGA‐HA@PLGA scaffold.

### Mechanical Characterization

The mechanical characteristics of PLGA, HA@PLGA, and FeGA‐HA@PLGA scaffolds were assessed using a uniaxial mechanical testing apparatus (Heng Yu Instrument Co., Ltd. HY‐940FS, Shanghai, China). Both the inner diameter and the depth of the casting mold were precisely set to 1.0 cm. Dimensions of diameter and height for each specimen were diligently measured and documented before the commencement of tests. Compression tests on the scaffolds were performed at a uniform strain rate of 2 mm per minute until failure was observed.

### Photothermal Properties of FeGA‐HA

The photothermal properties of FeGA‐HA‐3 h, FeGA‐HA‐6 h, and FeGA‐HA‐12 h nanowire were evaluated using NIR 808 nm, and temperature changes were recorded using an infrared thermal imager. The photothermal properties of FeGA‐HA nanowires were evaluated at a power density of 0.25 W/cm^2^ for 1 min.

### Photothermal Properties of PLGA, HA@PLGA, FeGA‐HA@PLGA scaffolds

The photothermal properties of three scaffolds were evaluated using NIR 808 nm, and temperature changes were recorded using an infrared thermal imaging camera. The photothermal properties of PLGA, HA@PLGA, FeGA‐HA@PLGA scaffolds were assessed at various power density(0.5 W cm^−2^, 1 W cm^−2^, 1.5 W cm^−2^, 2 W cm^−2^). The corresponding temperature stability of the FeGA‐HA@PLGA scaffolds was evaluated in five on/off cycles.

To determine the photothermal conversion efficiency (𝜂), the equation derived from the principle of thermal equilibrium was utilized.^[^ ^43^ ^]^

(2) $$\eta =\frac{\;\mathrm{hS}\left(T_{\max}-T_{\mathrm{surr}}\right)-Q_{0}}{I}$$

where *h* (%) was the heat transfer coefficient, *S* (cm^2^) was the surface area of the scaffold, *I* (W cm^−2^) was the effective sunlight power irradiated upon the scaffold, T_max_ was the equilibrium temperature of the test scaffolds and T_surr_ was the ambient temperature (the room temperature was set at 26.8 degrees Celsius and temperatures were recorded in degrees Celsius).

### In Vitro Antibacterial Assay

The spread plate method was employed to evaluate the antibacterial properties of different scaffolds against Gram‐negative *E. coli* (ATCC25922) and Gram‐positive *S. aureus* (ATCC25923). Five different groups were treated, including PLGA, HA@PLGA, HA@PLGA NIR, FeGA‐HA@PLGA, and FeGA‐HA@PLGA NIR, respectively. For the HA@PLGA NIR and FeGA‐HA@PLGA NIR groups, additional exposure to an 808 nm laser (1.5 W cm^−2^) was conducted for 10 min. The PLGA group was set as blank controls. In each group, scaffold specimens were immersed in 1 × 10^8^ CFU Ml^−1^ bacterial suspensions for 5 h. The bacterial solution was diluted to a concentration of 1 × 10^4^ CFU mL^−1^ using PBS. Subsequently, 100 µL of the diluted bacterial solution was spread onto LB agar and incubated at 37 °C. Then the bacterial colonies on the plates were examined after incubation for 16 h. The relative bacteria viability was assessed using the formula: Survival viability (%) = N_t_/N_c_ × 100%, where N_t_ denotes the colonies formed in the experimental group and N_c_ refers signifies colonies formed in the control group (PLGA group). The bacterial morphology on the different scaffolds was further investigated by SEM to evaluate the antibacterial effects. The co‐cultured supernatant and scaffold were fixed with glutaraldehyde, dehydrated with gradient ethanol, and dried in air. Subsequently, both the scaffold and supernatant were subjected to gold sputtering for SEM observation.

### NPN Uptake Assays

N‐phenyl‐1‐naphthylamine (NPN) was used to evaluate the permeability of the bacterial membrane.^[^ ^44^ ^]^ A 1 mM stock solution of N‐phenyl‐1‐naphthylamine (NPN) was prepared and then diluted to 40 µM with 30% DMSO to obtain the working solution. *E. coli* and *S. aureus* were cultured on a shaker for 24 h, after which 10^8^ CFU mL^−1^ were transferred into a 24‐well plate. PLGA, HA@PLGA, and FeGA‐HA@PLGA were introduced into the bacterial suspensions for photothermal or non‐photothermal treatment. Treated suspensions, 150 µL in volume, were added to a 96‐well plate and co‐incubated with 50 µL of the 40 µM NPN solution at 37 °C for 30 min. Fluorescence intensity at 420 nm was measured upon excitation at 350 nm.

### Cell Culture

Bone mesenchymal stem cells (BMSCs) were harvested from the femurs and tibias of two‐week‐old Sprague‐Dawley (SD) rats, acquired from SLAC (Shanghai, China). These BMSCs were propagated in α‐MEM supplemented with 10% fetal bovine serum (FBS, Gibco, USA) and 1% penicillin/streptomycin (PS, Gibco, USA), and maintained at 37 °C within a humidified 5% CO_2_ incubator. The culture medium was refreshed every 2–3 days, and cells reaching confluence were subcultured every 3–4 days following the established protocol. BMSCs from passages 3–5 were utilized in subsequent experiments.

### Cell Viability Test of Scaffolds

In Vitro: First, the sectioned small‐sized scaffolds were irradiated under ultraviolet light for 1 h, and then placed into α‐MEM culture medium supplemented with 10% FBS. After scaffold preparation, BMSCs were seeded at a concentration of 1 × 10^4^ cells per well in a 96‐well plate and cultured with α‐MEM for 24 h, and then put into the scaffolds and cultured for 24 h. The cells were transferred onto the prepared scaffolds and further incubated for an additional 24 h to facilitate cell‐scaffold interactions. The enhanced cell counting kit‐8 (CCK‐8, Beyotime Biotechnology, Jiangsu, China) was used to assess cell viability. Then, the optical density (OD) value of the reaction solution was measured using the microplate reader at a wavelength of 450 nm.

### Live/Dead Viability and Cellular Morphology on Scaffolds

In Vitro: First, the scaffolds were sterilized using ultraviolet irradiation, and BMSCs were then seeded onto the thin‐layer scaffolds at a density of 2 × 10^4^ cells per well within a 24‐well plate. After incubating for 24 h, cell viability among different treatment groups was evaluated using the live/dead staining kit (Beyotime Biotechnology, Jiangsu, China), following the provided protocol. The optical images of the live (green)/dead (red) staining of BMSCs cocultured with thin‐layer scaffolds were recorded using a fluorescence microscope (Olympus Corporation, Japan).

To characterize cellular morphology, a sterile thin‐layer scaffold was carefully placed at the center of a confocal dish and anchored with a sterile stainless steel ring to prevent displacement. Cells were then seeded onto the scaffold at a controlled density of 5 × 10^3^ cells per scaffold. After incubation for 3 days, the cell culture dishes were washed with PBS twice. Then, the scaffolds were fixed with 4.0% paraformaldehyde for 20 min and subsequently subjected to three washes with 0.1% Triton X‐100. For cytoskeletal visualization, Actin‐Tracker Red‐Rhodamine (Beyotime Biotechnology, China) was diluted by PBS solution (containing 3% of BSA and 0.1% of Triton X‐100). The scaffolds were incubated with diluted Actin‐Tracker Red‐Rhodamine solution at a 1:100 dilution for 40 min, followed by nuclear counterstaining with DAPI provided by Servicebio (Hubei, China) for a brief ten‐minute period. Subsequently, the cytoskeleton and cell nucleus of the cocultured BMSCs were stained by fluorescent Actin‐Tracker Red‐Rhodamine and DAPI, respectively. Optical imaging of the stained BMSCs was conducted with a confocal laser scanning microscope (ZEISS, LSM710, Germany).

### Hemolysis Assay

The 4% erythrocyte suspension was incubated with PLGA, HA@PLGA, FeGA‐HA@PLGA, and FeGA‐HA@PLGA NIR at 37 °C for 4 h in a cell culture incubator. Deionized water and PBS containing purified RBCs were used as the positive and negative controls, respectively. The samples were centrifuged at 1500 rpm for 10 min to pellet the blood cells. Subsequently, 100 µL of the sample supernatant without scaffold was carefully transferred to a 96‐well plate for further analysis. The absorbance attributable to hemoglobin present in the supernatant was quantified using a microplate reader set to a wavelength of 540 nm. The hemolysis radio of each sample was calculated using the following equation: Hemolysis% = (A_test_ − A_neg_)/(A_pos_ − A_neg_) × 100%, where A_test_, A_pos_, and A_neg_ were the absorbance values of the sample, and the positive and negative groups, respectively.

### Antioxidant Effect of Scaffold

In Vitro: The production of intracellular ROS (Reactive Oxygen Species) was measured using the fluorescent probe 2′,7′‐dichlorofluorescin diacetate (DCF‐DA). Before the assay, the BMSCs were seeded at a density of 2 × 10^4^ cells per well in a 6‐well plate. Then the different scaffolds (PLGA, HA@PLGA, FeGA‐HA@PLGA) were incubated with BMSCs for 48 h (37 °C, 5% CO_2_). After treatment, the cells were washed twice with PBS and then incubated with 10 µM DCF‐DA in serum‐free medium for 30 min at 37 °C in the dark. The DCF‐DA solution was then removed, and the cells were washed with PBS to eliminate any non‐internalized probe. ROS production was observed using fluorescence microscopy (ZEISS Axio Observer 3, Germany).

### Macrophages Polarization Assessment

In Vitro: To ascertain the effect of various scaffolds on macrophage polarization, raw 264.7 macrophages were incubated for 24 h, and then stimulated with lipopolysaccharide (LPS) at 100 ng mL^−1^ for 24 h. After removing the culture medium and washing twice with PBS, the cells were incubated with PBS, PLGA, HA@PLGA, and FeGA‐HA@PLGA for 48 h. The positive control for M2 macrophage polarization was treated with interleukin‐4 (IL‐4) at 20 ng mL^−1^. Detailly, raw 264.7 macrophages were collected and incubated with 100 µL PBS containing fluorochrome‐conjugated antibodies against CD86 (a marker for M1 macrophages) and CD206 (a marker for M2 macrophages) for 30 min at 4 °C. Flow cytometric analysis was conducted using a BD LSR Fortessa X‐20 flow cytometer. Results were subsequently analyzed using FlowJo analysis software.

### ALP Staining and ARS Staining

Alkaline phosphatase (ALP) and alizarin red S (ARS) staining assays were conducted to assess the osteogenic differentiation potential of BMSCs when co‐cultured with various scaffolds. BMSCs were seeded at a density of 5 × 10^4^ cells per well in a 24‐well plate and cultured for 24 h, and then the medium was changed by the α‐MEM containing scaffold with NIR or without NIR. The scaffold‐free group was set as the control group. After incubation for 7 and 14 days, the cells were washed thrice with PBS and fixed with 4% paraformaldehyde for 20 min. After fixation, the cells were subjected to an additional three washes with PBS to eliminate the remaining paraformaldehyde. For staining, the BCIP/NBT alkaline phosphatase color development kit and a 0.2% ARS staining solution (Solarbio, Beijing, China) were applied to the respective wells for 30 min according to the manufacturer's instructions. The stained cells were then visualized using an inverted fluorescence microscope. The quantitative analysis of ALP staining was carried out via Image J software (NIH, USA) by quantifying the stained areas.

### Transcriptome Sequencing and Data Analysis

RNA‐sequencing analysis was used to evaluate the expression of mRNA profiles in BMSCs for the HA@PLGA scaffold group and FeGA‐HA@PLGA scaffolds group. The RNA sequencing was used to explore the role of FeGA in regulating immune responses and osteogenic differentiation. Furthermore, the mRNA profiles in BMSCs were assessed to compare the photothermal‐treated group (FeGA‐HA@PLGA NIR group) with the non‐photothermal‐treated group (FeGA‐HA@PLGA group). BMSCs (5 × 10^6^ cells/mL) were co‐cultured with the different scaffolds as described above for 7 days and cultured in an osteogenic medium. Cells in the five groups were then lysed using TRIzol reagent (Ambion, Carlsbad, CA), and cell lysates were stored at ‐80 °C pending further analysis. Eukaryotic mRNA sequencing experiments used the Illumina TruseqTM RNA sample prep Kit method (Illumina Hiseq2000 platform, Majorbio Biotech, Shanghai, China) for library construction. The data were analyzed online with Majorbio Biotech cloud platform, and the cluster Profiler R package was utilized to conduct Kyoto Encyclopedia of Genes and Genomes (KEGG) pathway enrichment analysis, aimed at evaluating the molecular or biological functions of DEGs and identifying enriched metabolic pathways within DEGs compared to the entire genome background. Significance was determined by a Corrected P‐value < 0.05 for enrichment by DEGs.

### Western Blot Analysis

For Western Blot (WB) analysis, the BMSCs treated by various scaffolds (HA@PLGA and FeGA‐HA@PLGA) were lysed using RIPA lysis buffer (Beyotime, China) contained with proteinase and phosphatase inhibitors (APExBIO) for 15 min at 4 °C. Then, the mixture was centrifuged at 4 °C (13,000 rpm, 20 min), mixed with loading buffer (Beyotime, China), boiled for electrophoresis, and transferred to PVDF membranes (Millipore, USA). After the membranes were blocked with 5% BSA, the PVDF membranes were incubated with primary antibodies at 4 °C overnight and then washed three times by PBST (PBS with Tween). Next, incubated with secondary antibodies (Beyotime, China) for 1.5 h at room temperature. Ultimately, the membranes underwent processing with the Odyssey Scanning system, a product of Li‐Cor, USA, and subsequent analysis was performed using the Image Studio software.

### In Vivo Evaluation of Bone Regeneration of Femoral Defect Infected with Bacterial

All animal experiments were carried out according to protocols approved by the Animal Care and Use Committee of Shanghai Tenth People's Hospital, School of Medicine, Tongji University, Shanghai, China (Ethical approval number: SHDSYY‐2023‐44850102). Male Sprague Dawley (SD) rats (12 weeks, 240 g) were employed as the standard animal models. Before the surgery, the scaffolds (⊝ 3 mm × 4 mm) were contaminated with *S. aureus*. The rats were anesthetized, and the scaffolds contaminated with *S. aureus* were implanted into the cylindrical defects of the tibia. In the animal grouping, the photothermal therapy group was illuminated with NIR light (808 nm, 1.5 W cm^−2^) for 5 min each time once a week, continuing until the sixth‐week post‐treatment. After 2 weeks post‐implantation, 3 rats from each group were euthanized to evaluate bacterial infection at the implant sites. The remaining 8 rats in each group were sacrificed after 4 and 8 weeks post‐implantation, and the tibias with different implants were harvested and fixed before micro‐CT scanning and histological analysis.

### Micro‐CT

To evaluate the effect on bone regeneration, the obtained specimens were scanned and analyzed by the Micro‐CT system (Hiscan XM Micro CT). The X‐Ray tube was set to 80 kV and 100 µA, and images were captured at a resolution of 25 µm. Imaging involved a rotation step of 0.5 through a 360‐degree angular range, with each step having a 50 ms exposure. The cylindrical bone defect region (diameter: 3 mm and depth: 4 mm) was set as the region of interest for quantitative analysis of typical parameters. Trabecular bone parameters were assessed, encompassing bone mineral density (BMD, g cm^−3^), bone volume fraction (BV/TV, %), trabecular number (Tb. N, 1/mm), and trabecular thickness (Tb. Th, mm).

### Histology and Immunohistochemistry

After decalcification following the Micro‐CT scan, the samples were dehydrated in a graded ethanol series and embedded in paraffin. Then, sections of approximately 5 µm were obtained for further staining. First, H&E and Masson's trichrome staining were performed to evaluate bone formation and residual materials. Additionally, immunofluorescence staining (CD86 and CD206, IL‐6 and TNF‐α) evaluated the levels of inflammatory cytokines. Furthermore, immunofluorescence staining was carried out to evaluate the expression of osteogenic marker proteins (OCN). The microvessel density (MVD) was also evaluated by CD31 immunofluorescence staining. The quantification of MVD was determined by averaging the number of positive cells in three vascularized regions. Briefly, the deparaffinized sections were first blocked with 5% bovine serum albumin (BSA) solution, followed by incubation with primary antibodies against CD86, CD206, IL‐6, TNF‐α, CD31and OCN at a 1:100 dilution overnight at 4 °C. In addition, to assess the potential toxicity of implanted scaffolds in vivo, major organs, including the heart, liver, spleen, lung, and kidney, were collected at 6 weeks and stained with H&E. All samples were imaged with an optical microscope. The staining mentioned above collectively substantiates that the FeGA‐HA@PLGA NIR group reconstructs the osteogenic microenvironment in infected bone defects by modulating the immune milieu, promoting angiogenesis, and enhancing osteogenic differentiation, thereby facilitating bone regeneration.

### Statistical Analysis

All data were expressed as means ± standard deviation (SD). Statistical analysis was performed by one‐way analysis of variance (ANOVA) with post‐hoc Tukey's method for multiple comparisons. The values of *p < 0.05, **p < 0.01, ***P < 0.001, and ****P < 0.0001 for all tests were considered statistically significant.

## Conflict of Interest

The authors declare no conflict of interest.

## Supporting information



Supporting Information

## Data Availability

The data that support the findings of this study are available from the corresponding author upon reasonable request.
