# Supplementary material for: Biomimetic Scaffolds Regulating the Iron Homeostasis for Remolding Infected Osteogenic Microenvironment
Source: Adv Sci (Weinh). 2024 Oct 7;11(44):2407251. doi: 10.1002/advs.202407251 (PMC11600272; doi:10.1002/advs.202407251)
Supplement: Supplementary file 1 — Supporting Information [file ADVS-11-2407251-s001.docx]

**Biomimetic Scaffolds Regulating the Iron Homeostasis for Remolding Infected Osteogenic Microenvironment**

*Mengting Yin^1,a^, Zhiqing Liu^1,a^, Zhongyi Sun^a,b,c^, Xinyu Qu^a^, Ziyan Chen**^a^, Yuying Diao ^b^, Yuxuan Chen**g^a^,* *Sisi Shen^d^,* *Xiansong Wang^d^,* *Zhuyun Cai^a^,* *Bingqiang Lu^a^,* *Shuo Tan^a^,* *Yan Wang^*,b^,* *Xinyu Zhao^*, a^ and* *Feng Chen^*,a,b,c^*

^a^Center for Orthopaedic Science and Translational Medicine, Department of Orthopaedics, Shanghai Tenth People’s Hospital, School of Medicine, Tongji University Shanghai 200072, P. R China.

^b^Shanghai Key Laboratory of Craniomaxillofacial Development and Diseases Shanghai Stomatological Hospital & School of Stomatology, Fudan University, Shanghai 201102, P.R. China.

^c^Suzhou First People’s Hospital, School of Medicine, Anhui University of Science and Technology, Anhui 232001, P.R. China.

^d^Department of Plastic and Reconstructive Surgery, Shanghai Key Laboratory of Tissue Engineering, Shanghai Ninth People’s Hospital, Shanghai Jiao Tong University School of Medicine, Shanghai 200011, P.R. China.

^1^Authors have equally contributed to this work: Mengting Yin, Zhiqing Liu.

E-mail addresses:

xyzhao@tongji.edu.cn; kq_wangyan@fudan.edu.cn, chen_feng@fudan.edu.cn


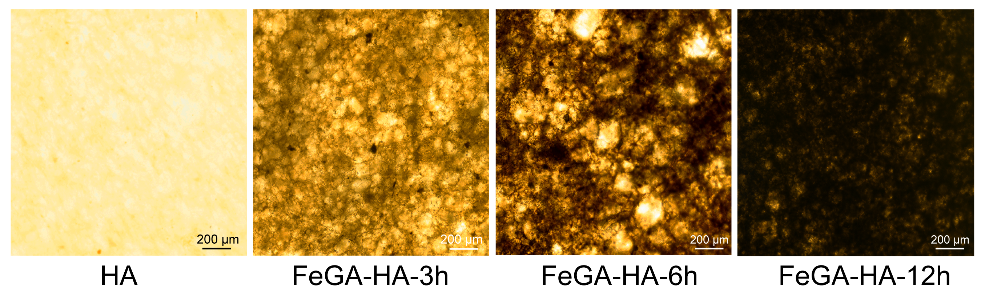


Figure S1. Optical images of HA and FeGA-HA prepared under different reaction times (Scale bar, 200 μm).

It can be observed that the longer the hydrothermal reaction time is, the more FeGA is enriched on the surface of the nanowires.


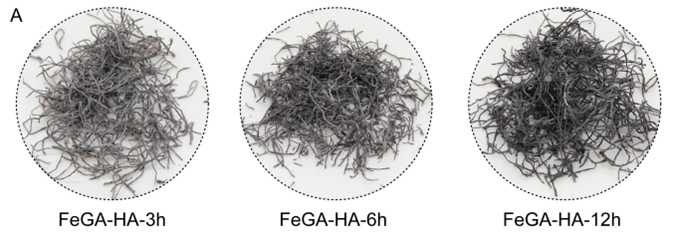


Figure S2. (A) The digital photos of FeGA-HA nanowires prepared under different reaction times.

With the increase in hydrothermal reaction time, the color of HA nanowires gradually changes from white to gray to dark gray and finally becomes black.


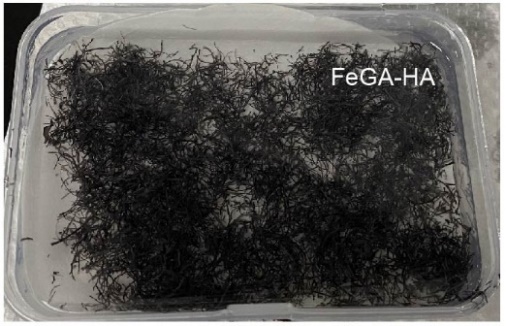


Figure S3. The FeGA-HA nanowires dispersed in anhydrous ethanol.

The modified HA can be uniformly dispersed in anhydrous ethanol and the suspension remains stable.


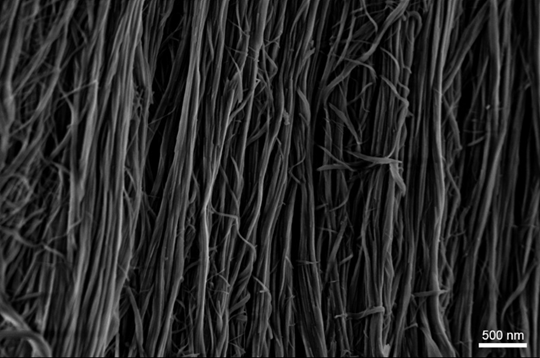


Figure S4. The SEM image of ultra-long hydroxyapatite nanowires.


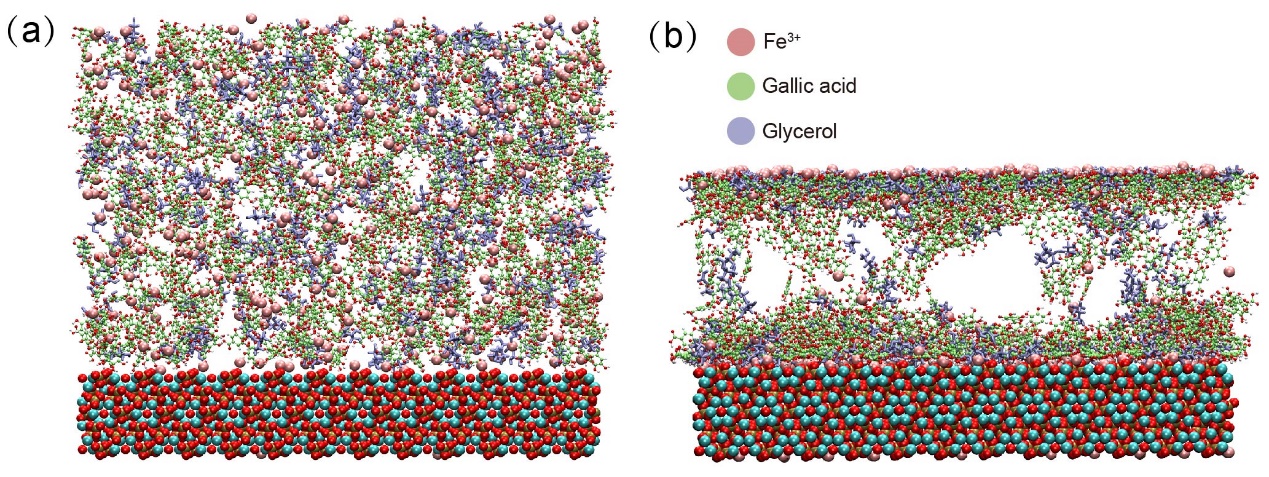


Figure S5. The adsorption of gallic acid and Fe^3+^ ions on HA surface before and after MD simulation. (a) HA, gallic acid, and Fe^3+^ ions in the complex system at 0 ns. (b) HA, gallic acid, and Fe^3+^ ions in the complex system at 50 ns.

Before the simulation of the system, the gallic acid and Fe^3+^ ions are randomly distributed in the solution. After 50 ns MD simulation, the gallic acid and Fe^3+^ ions are absorbed on the HA surface.


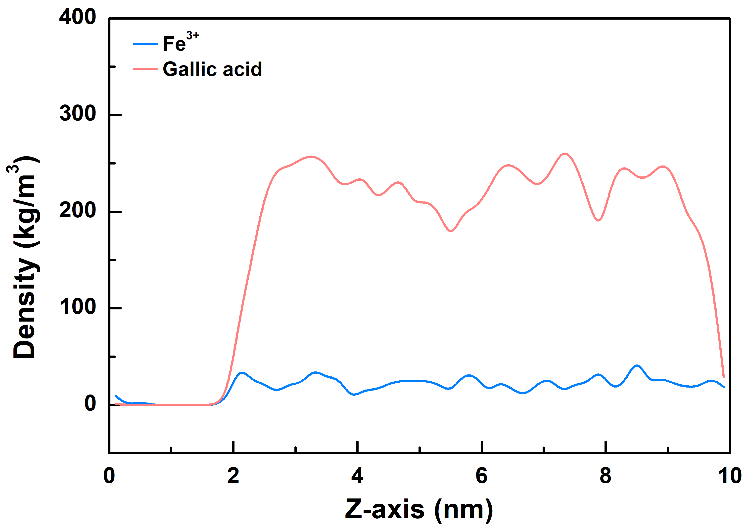


Figure S6. The distribution density of Fe^3+^ ions and gallic acid in the glycerol system.


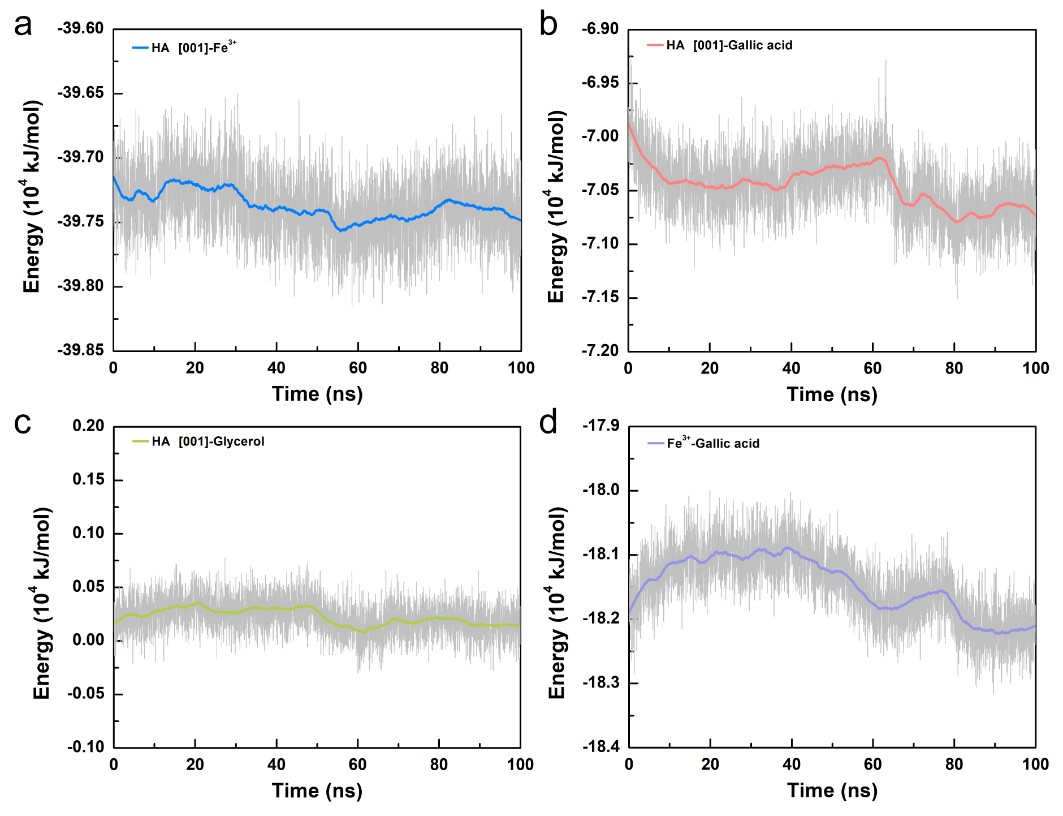


Figure S7. (a) The interaction energy between HA [001] and Fe^3+^ ions varies with time. (b) The interaction energy between HA [001] and gallic acid varies with time. (c) The interaction energy between HA [001] and glycerol varies with time. (d) The interaction energy between Fe^3+^ ions and gallic acid varies with time.


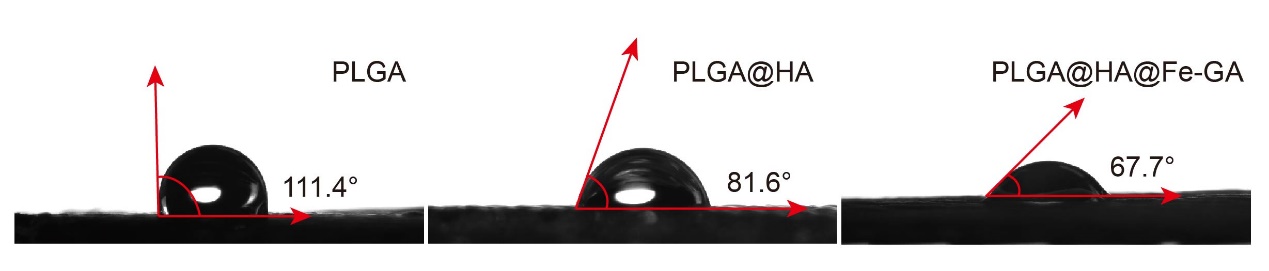


Figure S8. The contact angle of different scaffolds.

The contact angle of the FeGA-incorporated scaffold is smaller than those of the PLGA and PLGA@HA scaffolds.


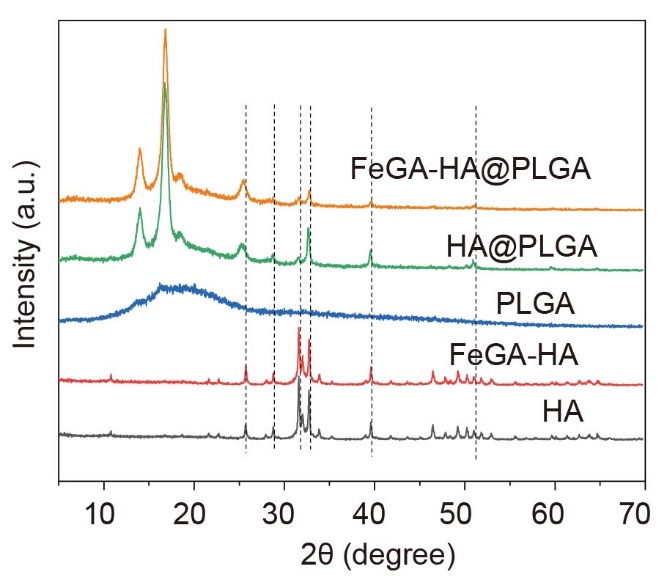


Figure S9. XRD patterns of HA, FeGA-HA, PLGA, HA@PLGA, and FeGA-HA@PLGA samples.


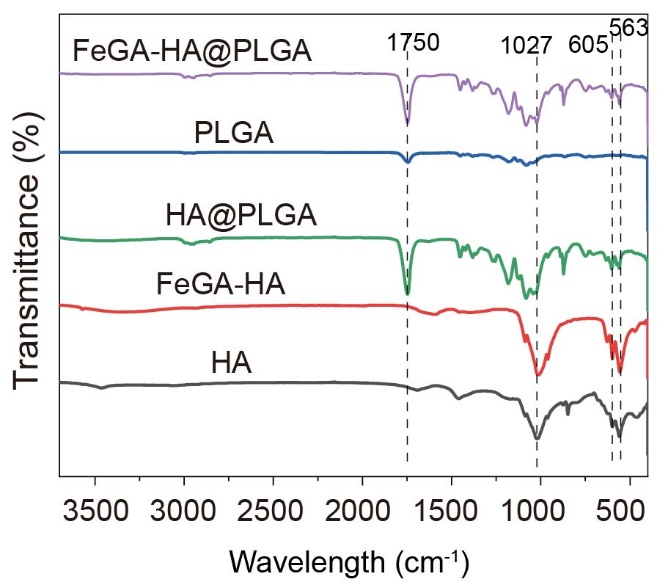


Figure S10. FTIR spectra of HA, FeGA-HA, PLGA, HA@PLGA, and FeGA-HA @PLGA samples.

The incorporation of FeGA-HA nanowires into the PLGA scaffold resulted in the crystallization of PLGA.


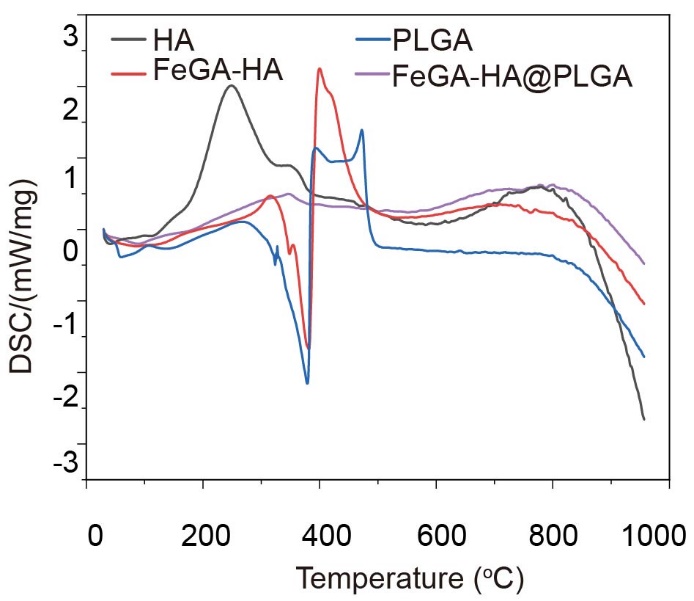


Figure S11. The differential scanning calorimetry analysis of HA, FeGA-HA, PLGA, and FeGA-HA@PLGA samples.


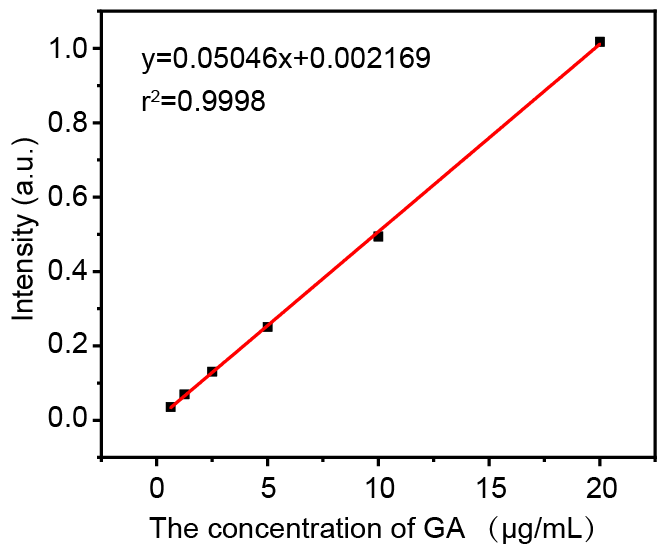


Figure S12. The standard curves of GA


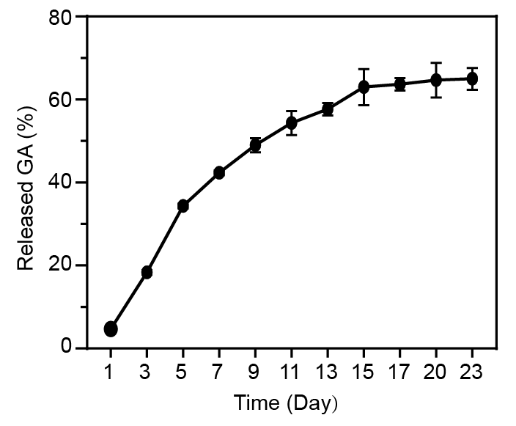


Figure S13. The sustained release curve of GA released from FeGA-HA@PLGA scaffold.

The result shows that benefitting from the biodegradability of PLGA, ~65% GA can be released from the FeGA-HA@PLGA scaffolds after 23 days in a simulated physiological environment.


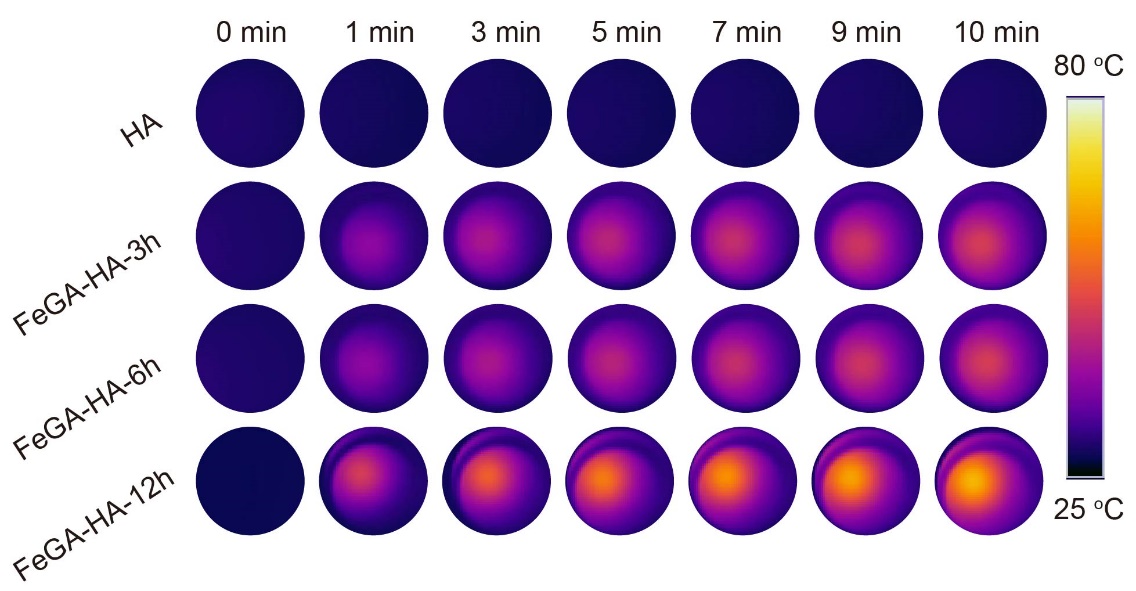


Figure S14. Infrared thermographic maps of HA, FeGA-HA-3h, FeGA-HA-6h, and FeGA-HA-12h with an NIR laser irradiation (808 nm, 0.5 W/cm^2^).

When the near-infrared light power is increased from 0.25 W/cm^2^ to 0.5 W/cm^2^, the FeGA-HA-12h nanowires wafer can be rapidly heated up to 67.7 ℃ within 1 min, while the temperature of FeGA-HA-3h and FeGA-HA-6h nanowires wafer only reach 51.8 ℃ and 59.1 ℃.


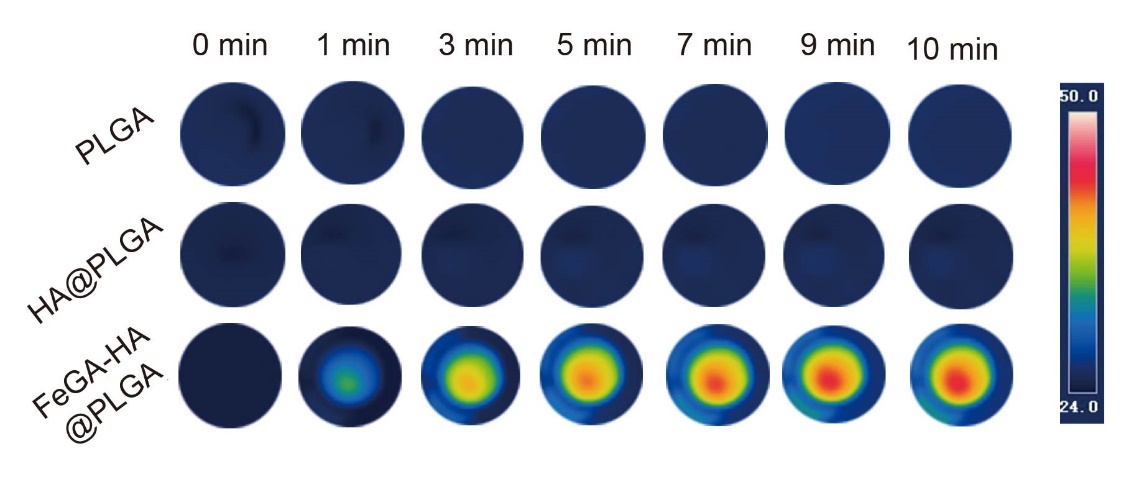


Figure S15. Infrared thermographic maps of PLGA, HA@PLGA, FeGA-HA@PLGA scaffolds with an NIR laser irradiation (808 nm, 1.5 W/cm^2^).

The infrared thermographic maps of the biomimetic bone repair scaffolds with different components at 1.5 W/cm^2^ show that the scaffolds loaded with FeGA-HA have the best photothermal performance.


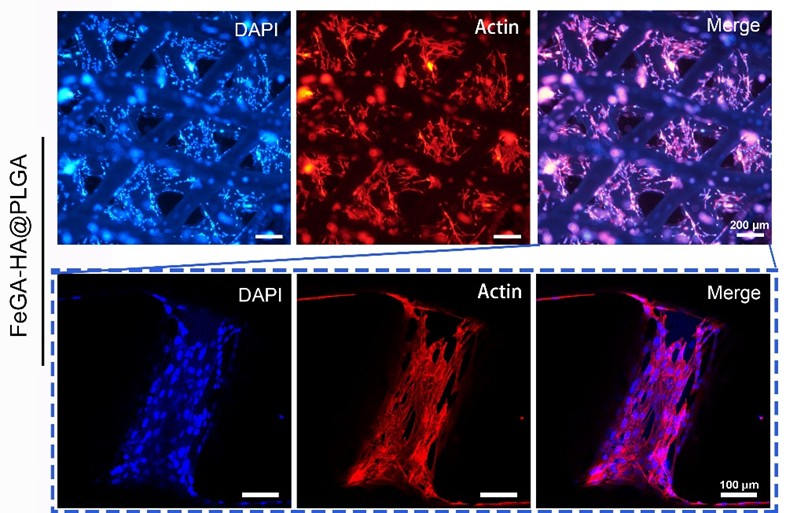


Figure S16. The actin staining of the cytoskeleton of BMSCs on FeGA-HA@PLGA scaffolds.

BMSCs are morphologically normal and grow well on FeGA-HA@PLGA scaffolds.


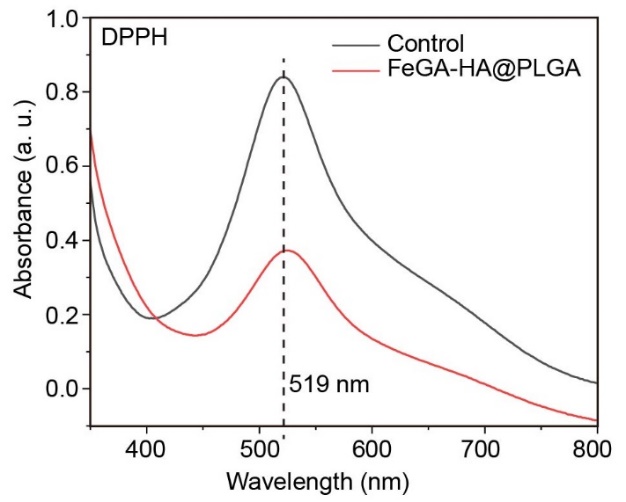


Figure S17. The scavenging capability of FeGA-HA@PLGA towards DPPH free radicals was assessed using UV-vis spectroscopy.


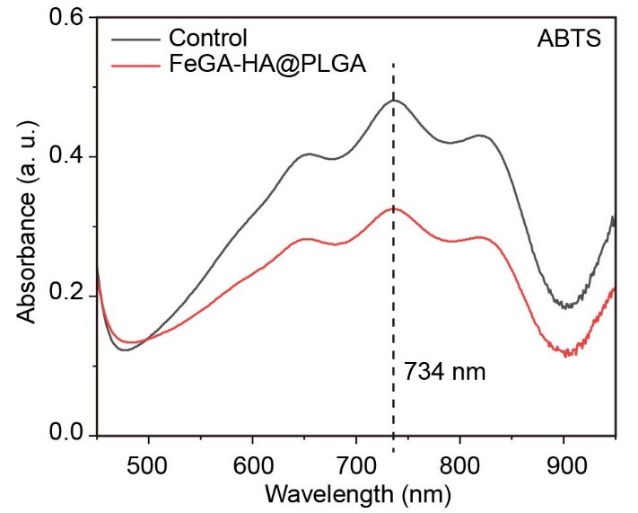


Figure S18. The scavenging capability of FeGA-HA@PLGA towards ABTS free radicals was assessed using UV-vis spectroscopy.


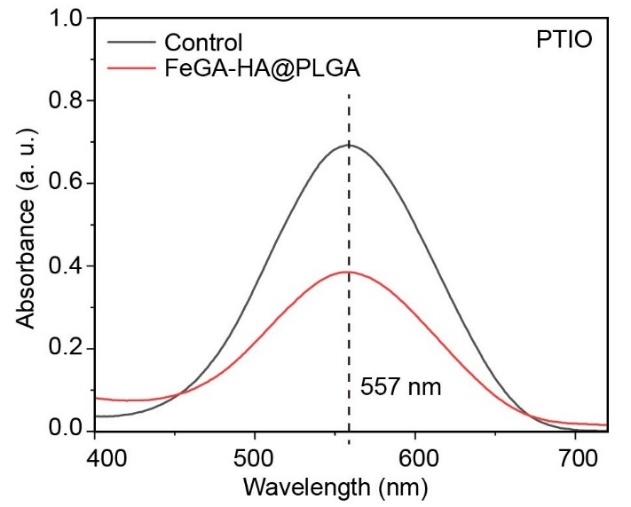


Figure S19. The scavenging capability of FeGA-HA@PLGA towards PTIO free radicals was assessed using UV-vis spectroscopy.

The FeGA-HA@PLGA scaffold can eliminate DPPH, ABTS and PTIO free radicals.


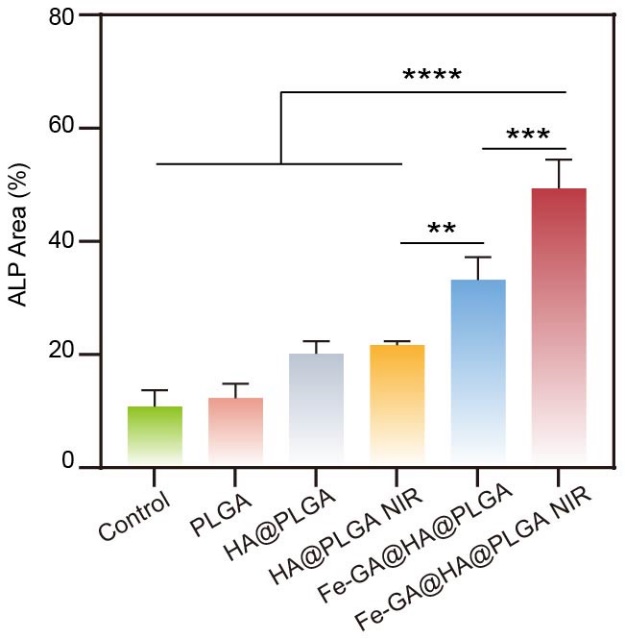


Figure S20. The corresponding quantification of ALP staining. The data were presented as the mean ± standard deviation (SD). *p < 0.05, **p < 0.01, ***p < 0.001, ****p < 0.0001.

After 7 days of cultivation, the FeGA-HA@PLGA group shows increased ALP activity compared to the PLGA and HA@PLGA groups, indicating that the FeGA-HA@PLGA scaffolds can promote the proliferation and differentiation of BMSCs.
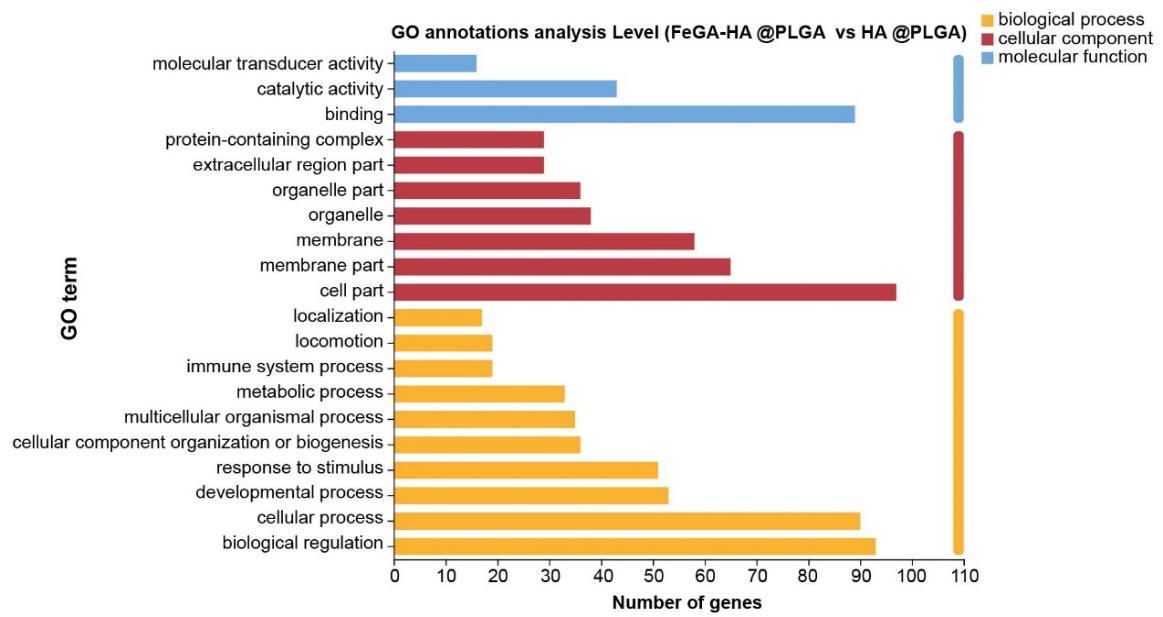


Figure S21. Gene ontology analysis of all genes in BMSCs cultured with FeGA-HA@PLGA or HA@PLGA scaffolds. BP, biological processes; CC, cellular components; MF, molecular functions.


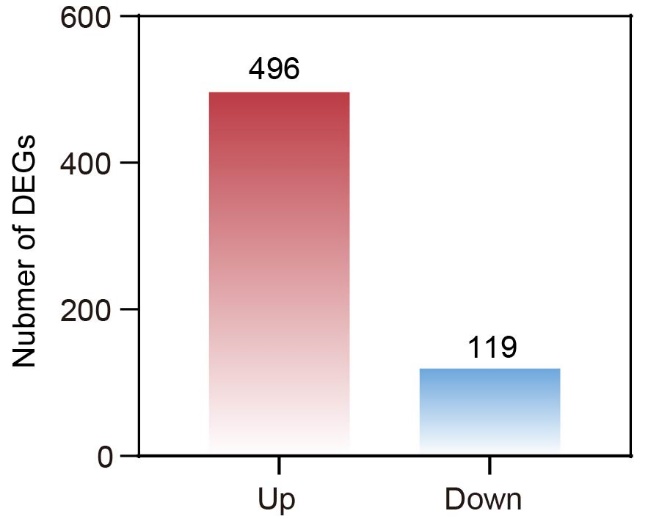


Figure S22. The number of upregulated and downregulated genes between FeGA-HA@PLGA NIR and HA@PLGA scaffolds.


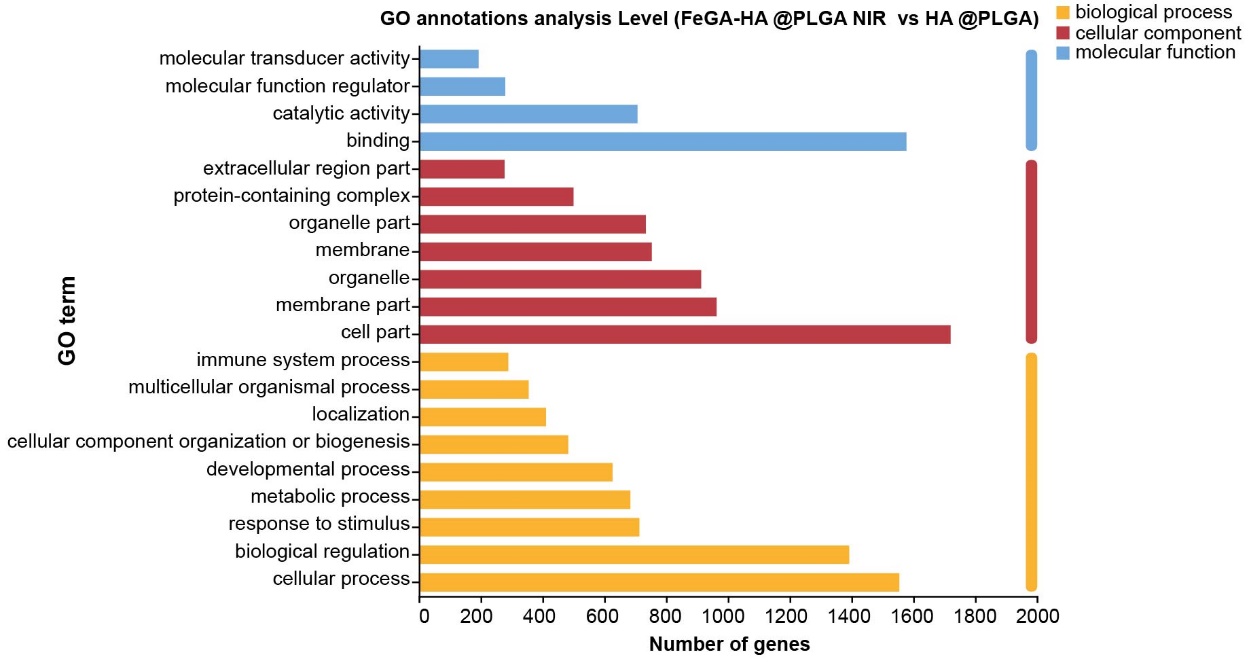


Figure S23. Gene ontology analysis of all genes in BMSCs cultured with FeGA-HA@PLGA NIR or HA@PLGA scaffolds. BP, biological processes; CC, cellular components; MF, molecular functions.


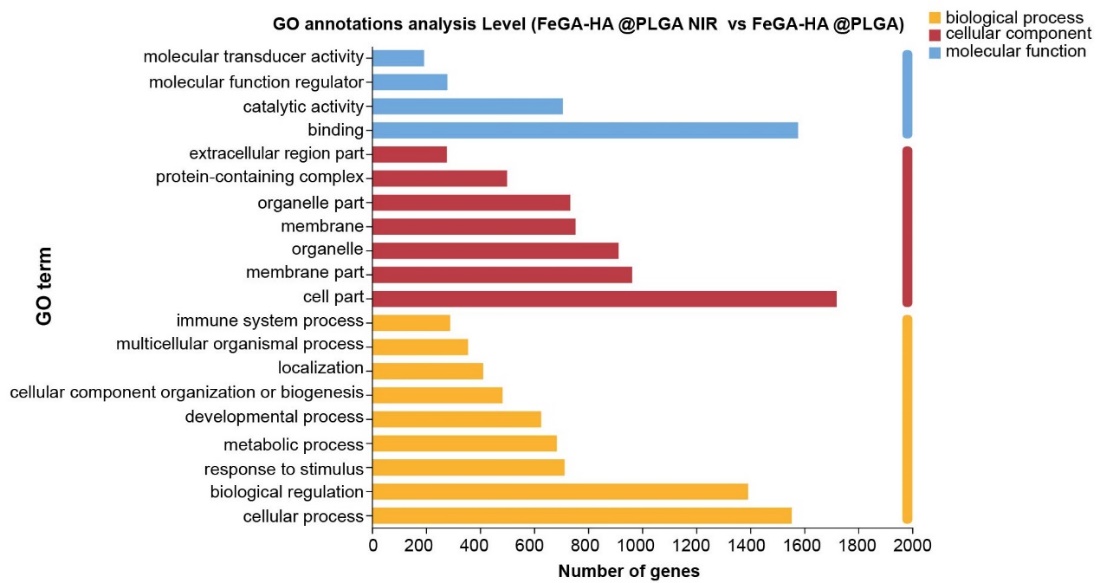


Figure S24. Gene ontology analysis of all genes in BMSCs cultured with FeGA-HA@PLGA NIR or FeGA-HA@PLGA scaffolds. BP, biological processes; CC, cellular components; MF, molecular functions.


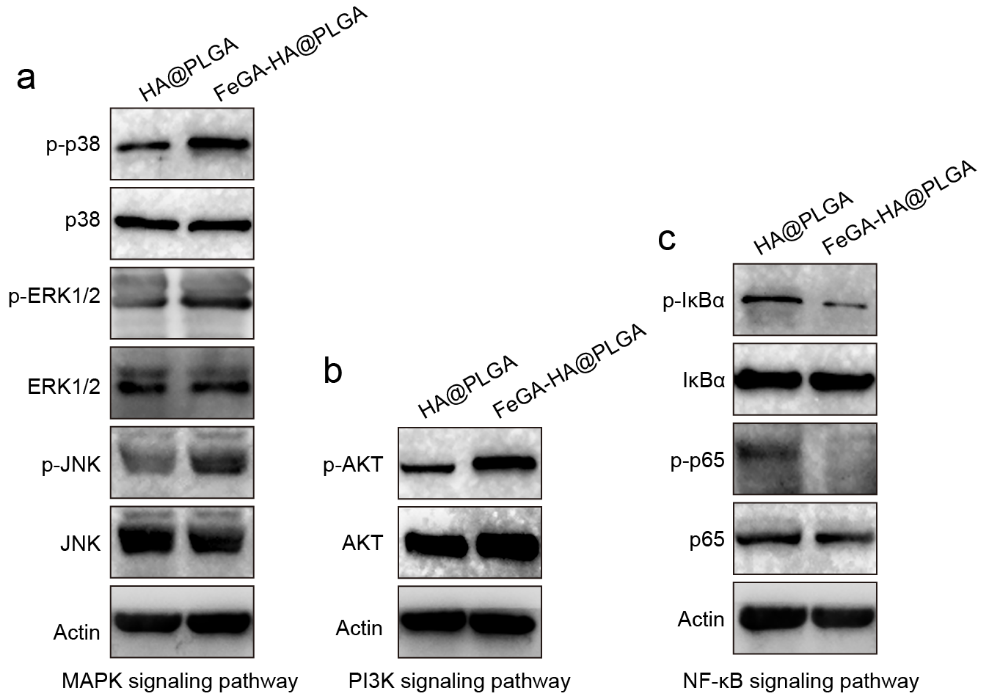


Figure S25. Western blot assay of the NF-κB, MAPK, and PI3K-AKT signaling pathways of BMSCs co-cultured with HA@PLGA and FeGA-HA@PLGA for 7 days.


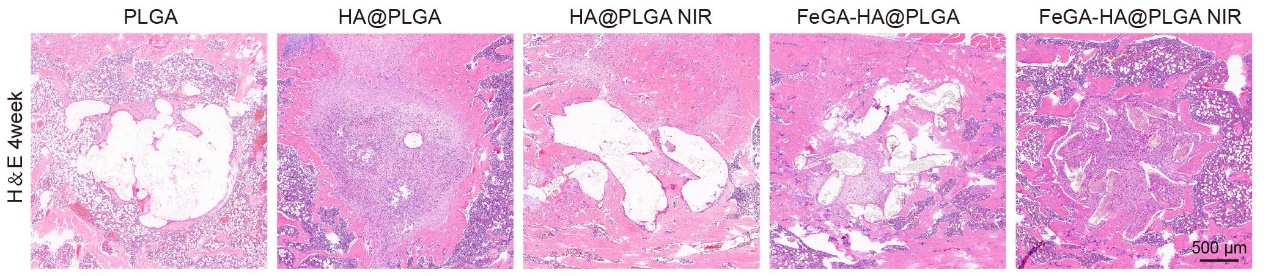


Figure S26. H&E staining of the PLGA group, HA@PLGA group, HA@PLGA NIR group, FeGA-HA@PLGA and FeGA-HA@PLGA NIR group at 4 week timepoint (scale bar: 500 μm).


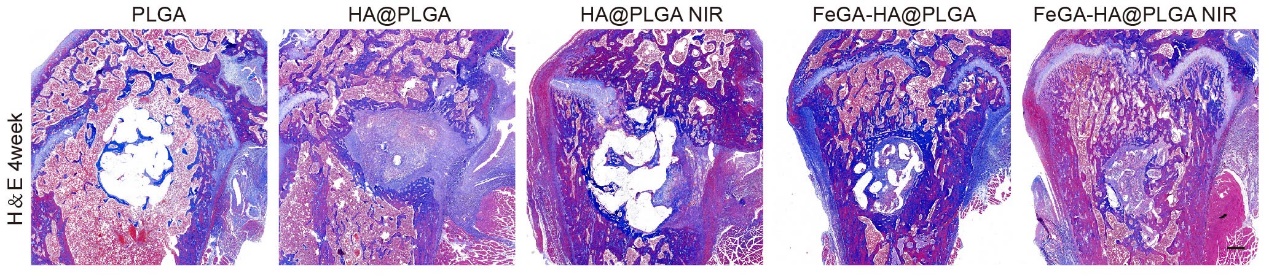


Figure S27. Masson's trichrome staining of the PLGA group, HA@PLGA group, HA@PLGA NIR group, FeGA-HA@PLGA and FeGA-HA@PLGA NIR group at 4 week timepoint (Scale bar: 500 μm).


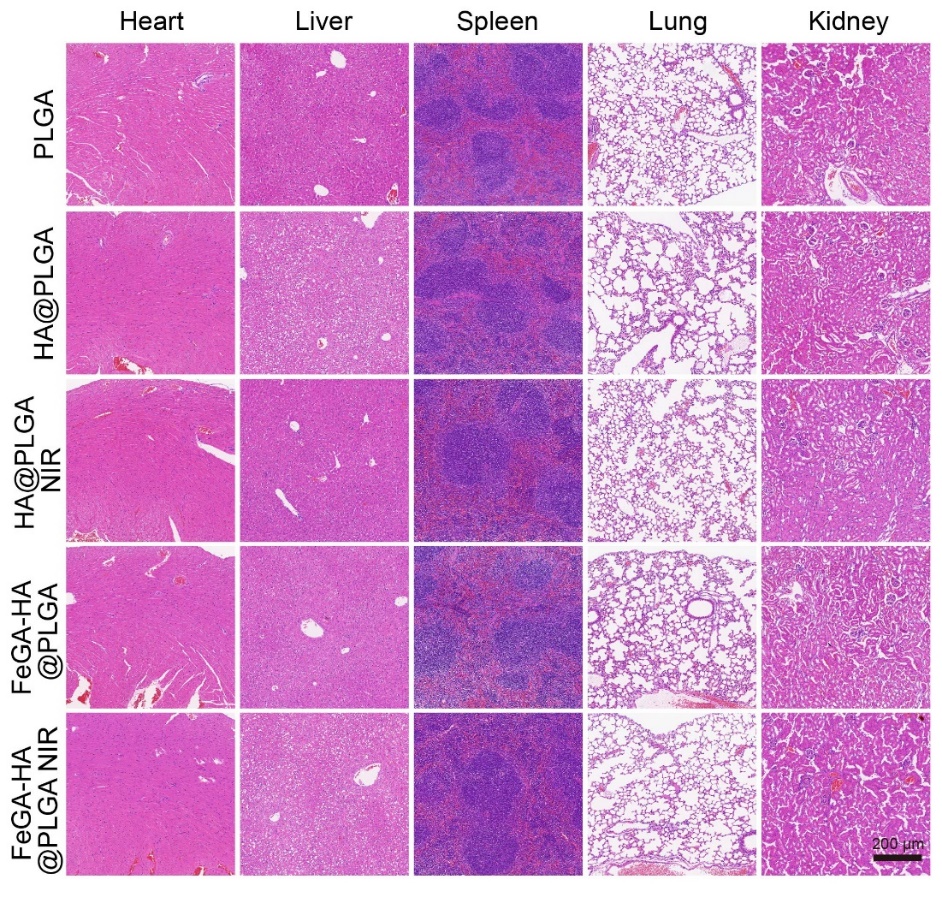


Figure S28.The H&E staining of heart, liver, spleen, lungs, and kidney, respectively (Scale bar: 200 μm)

The H&E staining results show that there are no obvious tissue damage and lesions in these five main organs, indicating that these scaffolds have good biological safety.
